# Induction of menstruation in mice reveals the regulation of menstrual shedding

**DOI:** 10.1101/2025.10.08.681007

**Published:** 2025-10-09

**Authors:** Çağrı Çevrim, Nicholas J. Hilgert, Aellah M. Kaage, Andrew J.C. Russell, Allison E. Goldstein, Claire J. Ang, Jaina L.R. Gable, Laura E. Bagamery, Ana Breznik, Daniela J. Di Bella, Mustafa Talay, Jingyu Peng, Kathleen E. O’Neill, Fei Chen, Sean R. Eddy, Kara L. McKinley

**Affiliations:** 1 Department of Stem Cell and Regenerative Biology, Harvard University, Cambridge MA, 02138, USA; 2 Department of Molecular and Cellular Biology, Harvard University, Cambridge MA, 02138, USA; 3 Broad Institute, Cambridge MA 02142, USA; 4 Biological and Biomedical Sciences Program, Harvard University, Cambridge MA, 02138, USA; 5 Howard Hughes Medical Institute, Cambridge MA 02138, USA; 6 Department of Systems Biology, Harvard University, Boston MA 02115, USA; 7 Department of Obstetrics and Gynecology, University of Pennsylvania, Philadelphia PA 19104

## Abstract

During menstruation, an inner layer of the endometrium is selectively shed, while an outer, progenitor-containing layer is preserved to support repeated regeneration. Progress in understanding this compartmentalization has been hindered by the lack of suitable animal models, as mice and rats do not menstruate. Here, we present transgenic mouse models that recapitulate the key anatomical, functional, and transcriptional features of human menstruation through targeted chemogenetic activation of premenstrual differentiation. Using single-cell spatial transcriptomics, we define a new paradigm for spatially regulated fibroblast differentiation that drives pre-menstrual endometrial layering and ultimately determines the extent of tissue shedding. Our results revise a century-old view of endometrial shedding and regeneration and establish new transgenic mice as powerful tools to advance menstruation research.

## Introduction

The uterine lining (endometrium) is classically thought to be organized into two functional layers: a superficial functionalis layer that cycles through differentiation, shedding, and regeneration, and a deeper basalis layer that remains undifferentiated throughout the cycle and is retained during menstruation (Critchley et al., 2020). The preservation of the basalis progenitor pool allows the endometrium to regenerate scarlessly, which is a unique example of complex organ regeneration in mammals. Damage to the basalis can disrupt endometrial regeneration and lead to endometrial scarring (Ang et al., 2023; Cousins et al., 2021; Salamonsen et al., 2021). Although this bilayered structure was described over a century ago (Sekiba, 1923), its cellular and genetic underpinnings remain poorly understood.

A major obstacle to studying menstrual biology is the lack of suitable animal models. Menstruation occurs in fewer than 2% of mammalian species. Ethical and technical constraints limit studies in the few species that naturally menstruate, and common laboratory animals such as mice and rats do not menstruate (Emera et al., 2012). This scarcity of models has greatly hindered progress in menstruation research.

**Figure 1.**
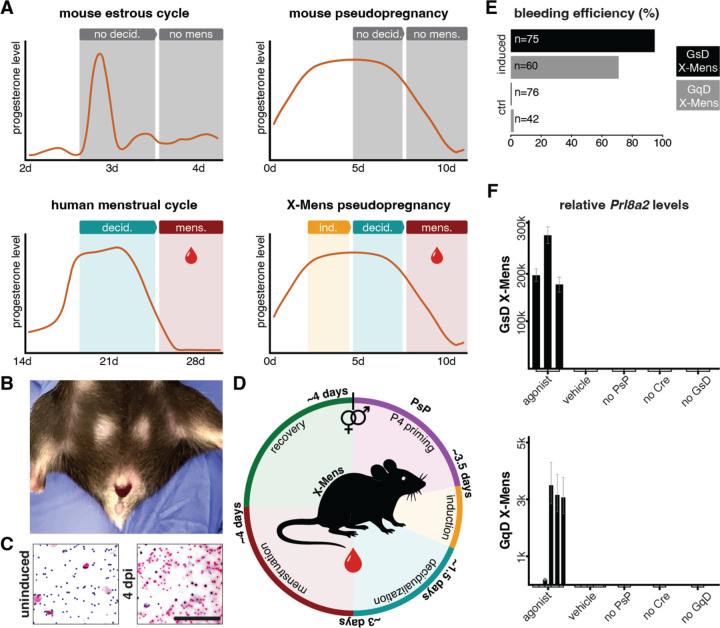
X-Mens: a transgenic mouse model of human-like menstruation **A.** Schematic overview of progesterone levels and major menstrual events in the post-ovulatory phases of wild-type mouse estrous and pseudopregnancy cycles, the human menstrual cycle, and the X-Mens pseudopregnancy cycle. d: days; decid: decidualization; mens: menstruation. **B.** Representative image of an X-Mens mouse exhibiting visible vaginal bleeding after induction and progesterone decline. **C.** H&E staining of vaginal lavage samples from X-Mens mice before induction (left) and during menstruation (right). Lavage samples consist of only epithelial cells and neutrophils before induction, whereas red blood cells dominate the composition 4 days post-induction, during menstruation (dpi). Scale bar: 200 μm. **D.** Schematic of the X-Mens menstrual cycle, starting with initiation of pseudopregnancy (PsP). P4: progesterone. **E.** Bleeding efficiency in GsD and GqD X-Mens mice following induction with agonist, as well as in control animals that did not receive agonist. **F.** Relative *Prl8a2* mRNA levels in X-Mens mice and genetic controls under indicated induction and pseudopregnancy conditions. Each bar represents the mean of the three technical replicates of one animal, and error bars indicate the standard deviation of those technical replicates.

The laboratory mouse (Mus musculus) provides a powerful opportunity to leverage extensive experimental resources for studies of the female reproductive tract (Cunha et al., 2019; Liu et al., 2020). Mice and humans share multiple anatomical similarities in their reproductive tracts and comparable cyclical changes in ovarian sex hormones (Cunha et al., 2019; Winkler et al., 2024). One striking difference is that, in humans, rising progesterone levels after ovulation induce decidualization, a cellular transformation of the endometrial fibroblasts that prepares the uterus for pregnancy. In non-pregnant cycles, progesterone levels fall, leading to degradation of decidualized tissue and ultimately to menstrual shedding. By contrast, in most non-menstruating mammals, including mice, decidualization occurs only upon embryo implantation and not during normal cycling; thus, endometrial shedding does not take place in non-pregnant cycles (Ramathal et al., 2010) (Figure 1A).

Intriguingly, decidualization can be induced in non-pregnant mice by experimental stimulation of the uterus through scratching or injection of vegetable oil into the uterine lumen (Buxton and Murdoch, 1982; Loeb, 1908). When progesterone levels fall after artificial induction of decidualization, the endometrium sheds and is expelled with overt vaginal bleeding (Finn and Pope, 1984). This mouse model recapitulates many aspects of human menstruation and has been instrumental in advancing our understanding of menstrual biology. For example, studies using this model have elucidated the roles of hypoxia, immune cells, sex hormones, and inflammation in endometrial shedding and regeneration, and enabled research into endometrial stem cells (Armstrong et al., 2017; Brasted et al., 2003; Greaves et al., 2014; Kaitu’u-Lino et al., 2007a; Kaitu’u-Lino et al., 2007b; Kaitu’u-Lino et al., 2010; Kirkwood et al., 2022; Liu et al., 2020; Maybin et al., 2018; Rogers et al., 2025; Shaw et al., 2022). However, important limitations remain. Induction of menstruation often requires major surgery, and animal welfare concerns limit the use of this approach for studying repeated cycles of menstruation—a core aspect of human uterine physiology. Most crucially, the procedure relies on non-physiological interventions that operate through unknown molecular mechanisms (Liu et al., 2020; Rogers et al., 2025).

To overcome these limitations, we established a transgenic system for inducible, repeatable menstruation in mice, leveraging relevant endometrial signaling pathways. Previous studies demonstrated that elevated calcium levels in endometrial epithelial cells and elevated cAMP in endometrial fibroblasts are important for mouse decidualization (Buxton and Murdoch, 1982; Ferrando and Nalbandov, 1968; Huang et al., 2014; Leroy and Lejeune, 1981; Rankin et al., 1977; Sakoff and Murdoch, 1994; Sakoff and Murdoch, 1996; Yu et al., 2020). By controlling endometrial calcium or cAMP levels with spatiotemporal precision, we induced decidualization and recapitulated core features of human menstruation. This approach enabled us to uncover how endometrial compartmentalization is regulated, and provides a new model for advancing our understanding of uterine physiology and menstrual health.

## Results

### Development of a transgenic method to induce human-like menstruation in mice.

We hypothesized that elevated calcium in the endometrial epithelium or elevated cAMP in endometrial fibroblasts would be sufficient to induce decidualization in progesterone-primed mouse endometrium. We generated two mouse strains with endometrial expression of engineered G-protein-coupled receptors known as DRE-ADDs (Designer Receptors Exclusively Activated by Designer Drugs), which can be selectively activated by their pharmacological agonists (Roth, 2016). In one strain, we controlled epithelial calcium levels by expressing Gq-DREADD (Zhu et al., 2016) with the epithelium-specific *Ltf-iCre* driver (Daikoku et al., 2014). We generated the second strain to control fibroblast cAMP levels by expressing Gs-DREADD (Akhmedov et al., 2017) in endometrial stromal cells with *Amhr2-Cre* (Jamin et al., 2002) (Supplementary text, Figure S1).

We induced a high-progesterone state in the transgenic mice through pseudopregnancy and systemically administered the DREADD agonists (referred to as agonist hereafter; see supplementary text). Three days post-induction (dpi) with agonist, both the Gq-DREADD and Gs-DREADD mouse strains exhibited vaginal bleeding (Fig. 1B–E). Bleeding occurred approximately 7.5 days after the onset of pseudopregnancy, when progesterone levels begin to decline due to the demise of corpora lutea (luteolysis) (Atkinson and Hooker, 1945; Rudolph et al., 2012), akin to the luteolysis-associated progesterone decline that triggers human menstruation. Vaginal bleeding lasted for approximately 4 days, and the induction protocol could be repeated as early as 4 days after bleeding cessation (Fig. 1D). These experiments demonstrate that chemogenetic elevation of endometrial calcium or cAMP along with a rise and fall of progesterone reliably induces overt vaginal bleeding in mice, creating two transgenic models for induction of menstruation on demand. We name the DREADD-based menstruation approach **Ch**emogenetic **I**nduction of **Mens**truation (**Χ-Mens**) and refer to the strains based on their respective DREADDs: GqD X-Mens and GsD X-Mens.

In humans, the duration of menstrual bleeding varies substantially, both between individuals and across different cycles in the same individual (Wang et al., 2024). Similarly, in both X-Mens strains, the onset and duration of menstrual bleeding varied, despite the animals having a common genetic background and the use of a standardized treatment protocol (Figure S2A and B). To assess intra-individual variability, we leveraged the capacity of our models to undergo repeated cycles of menstruation. Variability was evident within individual mice undergoing successive cycles of menstruation (Figure S2C and D). We hypothesized that this variation arose from differences in the timing of progesterone decline toward the end of pseudopregnancy. To investigate whether menstrual variation in X-Mens was hormonally regulated, we administered Mifepristone, a progesterone receptor antagonist, to GsD X-Mens mice at 2 dpi. This intervention markedly reduced variability, with all animals initiating bleeding at 3 dpi (1 day after Mifepristone administration) and most exhibiting bleeding for only one day (Figure S2B). These results demonstrate that X-Mens mice undergo vaginal bleeding associated with progesterone decline, as occurs in humans, and are amenable to studies of repeated menstruation.

**Figure 2.**
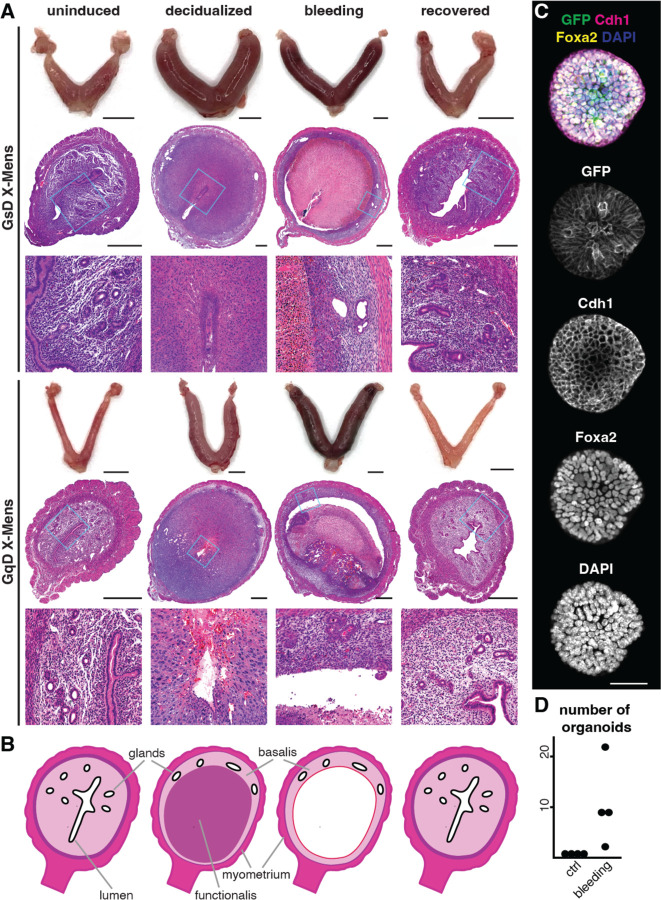
Human-like changes in the endometrium of menstruating X-Mens mice. **A.** Representative images of uteri and H&E-stained cross sections from X-Mens mice. Insets: uninduced morphology with intact luminal epithelium and glands spanning the full endometrial thickness. At peak decidualization (2 dpi), glands are displaced basally, and tissue exhibits increased vascularization, polyploidy, and cell density. During bleeding, the decidual layer detaches from the outer endometrium, with red blood cells infiltrating the tissue, indicating vascular breakdown. One-week post-menstruation, uterine morphology resembles the uninduced state with an intact lumen, appropriately distributed glands, and absence of decidual cells or red blood cell infiltration. Scale bars are 5mm for whole-mount uteri images, and 500μm for uterine cross sections. The scale bars demonstrate the significant enlargement of uteri following induction. **B.** Schematic illustration of the compartments of decidualized and menstruating X-Mens uteri. **C.** Representative immunofluorescence images of an organoid derived from menstrual effluent of GsD X-Mens mice. Scale bar: 50 μm. **D.** Quantification of endometrial organoids per well from control and menstruating animals.

We next assessed the extent to which menstruation in the X-Mens strains resembled human menstruation. Bleeding through vagina is characteristic of menstruation, but can also occur independently of menstruation, such as during ovulation (heat) in dogs and cattle (Hansel and Asdell, 1952; Sato et al., 2016), as well as in some endometrial pathologies including cancer (Wouk and Helton, 2019). Therefore, we sought to determine whether the two X-Mens strains exhibited other hallmarks of menstruation, including decidualization before bleeding and extensive shedding of live endometrial cells. We assessed decidualization by analyzing the expression of the well-established decidual marker, prolactin gene *Prl8a2* in 2 dpi uteri, approximately 1 day prior to bleeding onset (Orwig et al., 1997). Quantitative PCR analysis revealed that *Prl8a2* was upregulated in both X-Mens strains following agonist treatment but not in controls lacking agonist exposure. Administration of agonist to animals that were not pseudopregnant, or to genetic controls carrying only one of the required transgenes (Cre or DREADD), did not induce *Prl8a2* upregulation (Figure 1F). Similarly, pseudopregnant animals treated with agonist exhibited an increase in uterine volume (Figure 2A), another recognized hallmark of decidualization in mice. This growth response, like *Prl8a2* expression, was specific to experimental animals that received agonist during pseudopregnancy and was absent in controls (Figure S3), demonstrating the specificity and hormonal dependency of the X-Mens approach.

Histological analysis of 2 dpi uteri revealed tissue and cell morphologies characteristic of decidualization (Ramathal et al., 2010), including pronounced decidual cell differentiation, extensive vascularization, and displacement of endometrial glands toward the deeper layers of the endometrium (Figure 2A and B). Red blood cells infiltrated the decidual tissue, indicating localized vascular degradation and tissue breakdown consistent with menstrual shedding (Figure 2A). With the onset of bleeding, the decidualized layer began to detach from the underlying non-decidualized tissue and was shed into the uterine lumen. To confirm that live endometrial tissue was shedding during menstruation, as occurs in humans, we collected menstrual effluent and cultured the collected cells according to established endometrial organoid protocols (Turco et al., 2017). Organoids developed from lavages obtained during periods of vaginal bleeding but not from samples collected before induction, confirming live endometrial tissue sheds exclusively during menstruation (Figure 2C and D). Together, the X-Mens models exhibit premenstrual decidualization and hormone-dependent endometrial shedding.

Post-menstrual GsD and GqD X-Mens mice displayed uteri comparable in size and appearance to uninduced controls. Endometrial morphology returned to its pre-induction state, characterized by a continuous epithelial monolayer, normalization of endometrial gland distribution, and the absence of decidual cells (Figure 2A). At this stage, we were able to repeat the X-Mens protocol up to five times (Figure S2C and D), enabling successive cycles of menstruation and demonstrating that the endometrium retains its capacity for decidualization. We also confirmed pregnancy in post-menstruation animals, indicating functional recovery of the regenerated endometrium (supplementary text, Figure S4). These results demonstrate that the X-Mens mice exhibit both anatomical and functional regeneration of the endometrium following menstruation.

Both X-Mens strains exhibit endometrial changes characteristic of human menstruation, but there are important biological and practical distinctions between them (supplementary text). A key distinction lies in how the decidualization signal is relayed to the endometrium. In GsD X-Mens mice, all endometrial fibroblasts express GsD, and systemic delivery of the agonist initiates decidualization uniformly throughout the endometrium, closely mirroring the effect of circulating progesterone in humans. By contrast, GqD X-Mens mice rely on epithelial signaling to induce decidualization, as occurs during rodent pregnancy and in the conventional oil-induced model. Based on its similarity with human menstruation, we selected the GsD-X-Mens strain for additional interrogation.

### Transcriptional and architectural changes in the X-Mens endometrium mirror human menstruation.

Following induction, the endometrium of both GsD and GqD X-Mens strains exhibited histologically distinct superficial and basal layers. Subsequently, a plane of separation formed between these layers, leading to detachment and shedding of the superficial layer and leaving behind non-decidual fibroblasts and glandular epithelial cells (Figure 2A and B, Figure S5). This endometrial compartmentalization was strikingly similar to that observed in humans, in which the basalis layer comprised of fibroblasts and remnant glands persists during menstruation (Cousins et al., 2021; Salamonsen et al., 2021; Sekiba, 1923).

**Figure 3.**
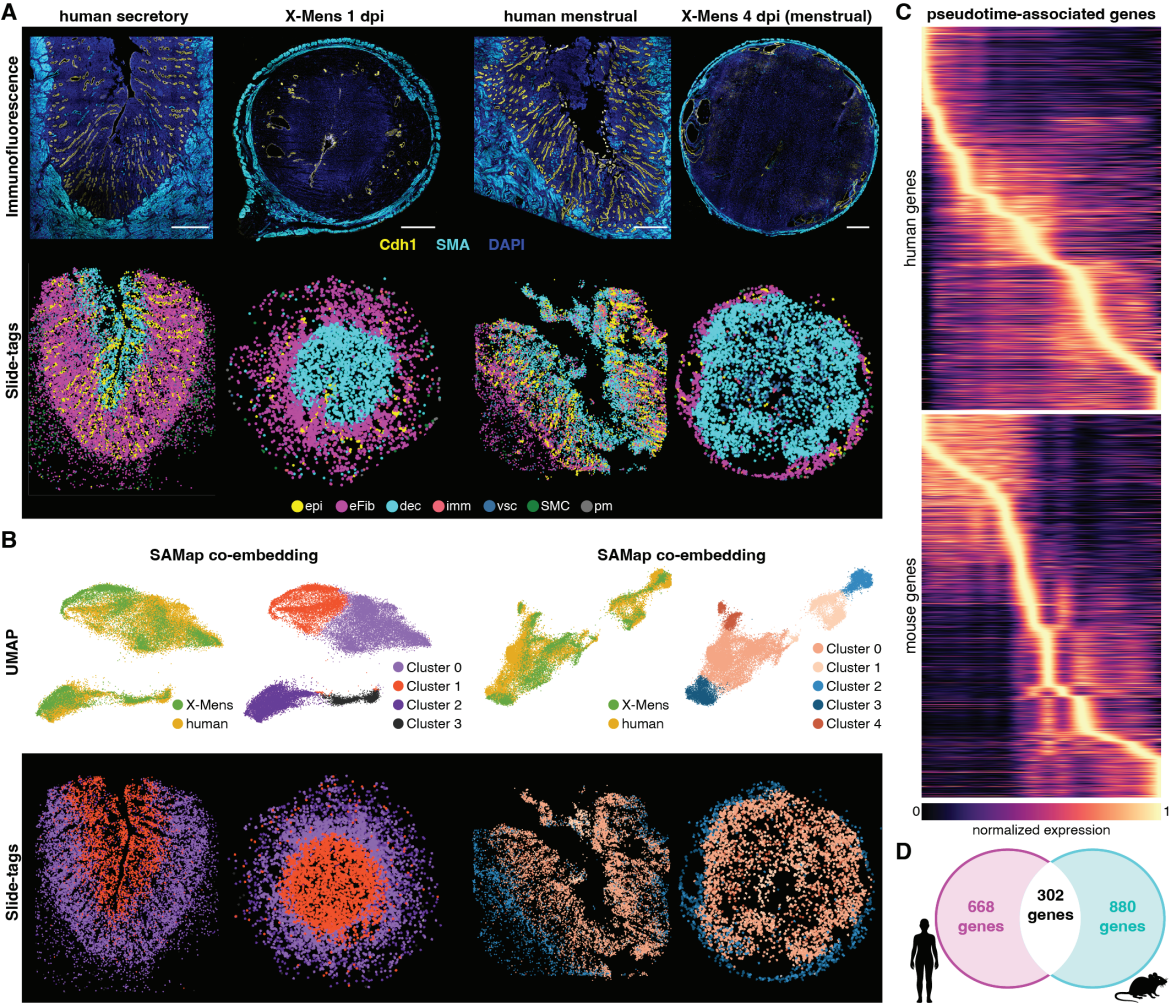
Homologous layering in human and X-Mens endometrium. **A.** Top row: immunofluorescence images of cross sections from decidualized and menstruating human and X-Mens uteri, stained for epithelium (Cdh1, yellow) and myometrium (SMA, cyan); nuclei are marked with DAPI (blue). Bottom row: spatial mapping of all cell types for each sample. The immunofluorescence staining and Slide-tags analysis were performed on consecutive tissue sections from each sample. Scale bars: 1mm. Epi: epithelial cells; eFib: endometrial fibroblasts; dec: decidual cells; imm: immune cells; vsc: vasculature cells; SMC: smooth muscle cells: pm: perimetrium cells. **B.** UMAP and spatial mapping of endometrial fibroblasts (eFib) and decidual cells (dec) co-embedded using SAMap. The left two panels show decidualized samples, and the right two panels show menstrual samples. **C.** Heatmaps showing the normalized expression of pseudotime-associated genes along the pseudotime in human (top) and mouse (bottom) samples. **D.** Venn diagram showing the overlap of pseudotime-associated genes with one-to-one orthologs among human and X-mens mouse. Human-specific genes are shown in magenta, X-Mens mouse-specific genes in cyan, and shared genes in white.

To determine whether the parallels in endometrial compartmentalization extended to the molecular level, we employed comparative spatial transcriptomics on samples from humans and X-Mens mice. We performed Slide-tags spatially resolved single-nucleus RNA sequencing (Russell et al., 2024) on full-thickness endometrial samples, which included the entire depth from the luminal surface to the myometrium, from GsD-X-Mens mice and two human donors without known reproductive diseases. For each species, one sample represented the decidualized state (secretory phase in human and 1 dpi in mouse) and the other represented the menstrual phase (4 dpi in mouse) (Figure 3A). After confirming the presence of all major endometrial cell types in both species (supplementary text, Figure S6 and S7), we focused further analysis on the endometrial fibroblast lineage, the key cells undergoing decidualization.

To assess the similarities in cell types and their positions between species, we co-integrated endometrial fibroblast and decidual cell datasets from both species into a common embedding using SAMap (Tarashansky et al., 2021). SAMap uses both sequence similarity and transcriptional nearest-neighbor relationships to infer cell type homology across species. We performed cross-species integration separately for cells from decidualized samples and from menstrual samples, resulting in partitions of the integrated data into four and five clusters, respectively. Each cluster contained cells from both species. Spatial mapping revealed one cluster that localized to the luminal side of the endometrium and a second cluster that localized to the basal side in both humans and mice, consistent with the inner-outer layering associated with the functionalis and the basalis (Figure 3B, S8 and S9). These findings indicate that X-Mens mice faithfully recapitulate the molecular signatures and spatial organization of inner and outer layers characteristic of the human endometrium.

To further assess the transcriptional similarity between the X-Mens model and human pre-menstrual differentiation, we combined the human decidual and menstrual samples into one dataset, and the mouse decidual and menstrual samples into another. We performed diffusion pseudotime analysis in each species, which orders cells based on transcriptomic similarity (Nayak and Hasija, 2021). This analysis produced a trajectory that extended from endometrial fibroblasts to decidual cells in both species (Figure S10). To determine whether fibroblast-to-decidual cell differentiation is governed by shared genetic programs, we identified genes associated with pseudotime progression for each species (Figure 3C). Both species-specific and shared gene lists contained established decidualization markers. These markers included *IGFBP1*, *LEFTY2*, and *IL11* in humans and *Hand2*, *Wnt4*, and *Bmp2* in X-Mens mice. Decidualization markers shared between both species included *Cebpb*, *Foxo1*, and *Wnt5a* (supplementary data S1). Overall, 31% of the top pseudotime-associated genes in humans were shared with X-Mens mice (Fig. 3D), a proportion significantly higher than the 12% overlap expected if decidualization and menstruation were governed by divergent genetic programs in the two species (hypergeometric test; p ≈ 3.2 × 10^−63^; see methods). Thus, X-Mens mice recapitulate key genetic programs of human pre-menstrual differentiation.

### The functionalis consists of layers of cells at different stages of decidualization.

Although compartmentalization of the endometrium into functionalis and basalis has been recognized for over a century (Sekiba, 1923), our understanding of this phenomenon remains limited. This is largely due to the scarcity of full-thickness peri-menstrual tissue from human patients and substantial inter-individual variation arising from genetic and life history differences. By contrast, the X-Mens model enables highly reproducible studies, as the animals are isogenic and raised in uniform conditions with matched reproductive histories. Having established that X-Mens faithfully recapitulates the human-like functionalis–basalis structure, we next used this model to dissect the mechanisms underlying this compartmentalization.

**Figure 4.**
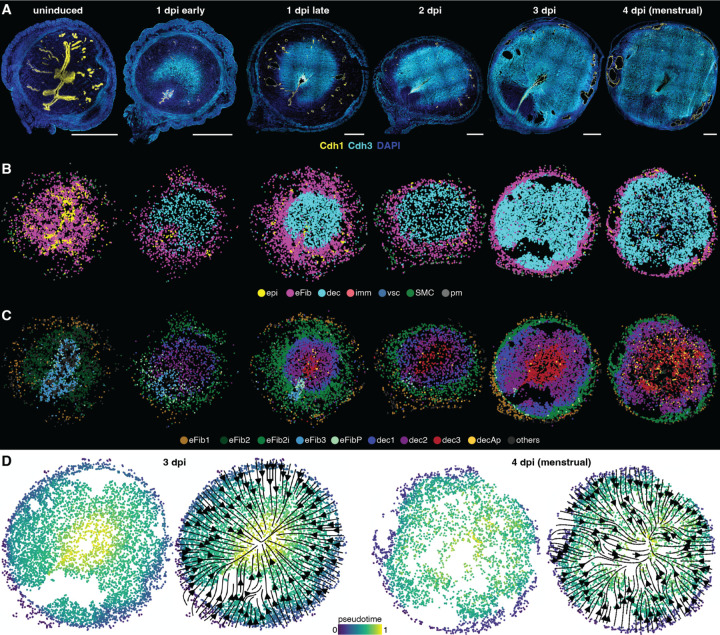
Multilayered compartmentalization in the menstruating X-Mens endometrium. **A.** Immunofluorescence images of GsD X-Mens uterine cross sections at sequential stages of the menstruation protocol, stained for epithelium (Cdh1, yellow) and decidual cells (Cdh3, cyan). DAPI marks nuclei (blue). Scale bars: 1mm. The 1 dpi late and 4 dpi menstrual sections are from the same uteri as in Figure 3. **B.** Spatial mapping of cell types at each stage. **C.** Spatial mapping of subtypes of endometrial fibroblasts (eFib) and decidual (dec) at each stage. Epi: epithelial cells; eFib: endometrial fibroblasts; dec: decidual cells; imm: immune cells; vsc: vasculature cells; SMC: smooth muscle cells: pm: perimetrium cells; eFib2i: endometrial fibroblasts 2 induced; eFibP: proliferating endometrial fibroblasts; decAP: apoptotic decidual cells. **D.** Spatial mapping of eFib and dec cells from 3 dpi (left) and 4 dpi menstrual (right) samples, colored by pseudotime values with or without pseudotime gradient streamlines indicating the direction of differentiation.

To characterize the dynamics of endometrial compartmentalization, we expanded our GsD X-Mens Slide-tags dataset by sampling additional time points from pre-induction through the onset of bleeding. Using Cdh3 (P-cadherin), a marker for decidual cells (Kadokawa et al., 1989) (Figure S11), we performed immunofluorescence to assess the extent of decidualization in each animal (Figure 4A). Based on these analyses, we selected six key stages—uninduced, decidualized (early 1 dpi, late 1 dpi, 2 dpi, 3 dpi), and menstrual (4 dpi)—for comprehensive Slide-tags profiling (Figure 4A, S6). Cell types identified based on marker gene expression changed over time in accordance with immunofluorescence on adjacent sections: endometrial fibroblasts gradually decreased over time, while decidual and vascular cell populations gradually increased as the cycle progressed (Figure S6, 4B).

We focused on the endometrial fibroblast (eFib) and decidual cell populations and performed Leiden sub-clustering, which identified nine clusters: five eFib and four decidual subtypes. Spatial mapping showed that each subtype occupied a unique position within the endometrium (Figure 4C, Figure S12). At all time points, the eFib1 subtype localized to the basal region of the endometrium, bordering the myometrium. The eFib3 population was initially situated beneath the luminal epithelium but diminished as decidualization progressed. eFib2 occupied the region between eFib1 and eFib3 in the uninduced time point. In induced samples, a distinct but closely related cluster occupied the same position, which we named eFib2 induced (eFib2i; see supplementary text). We also identified a small proliferating fibroblast subtype (eFibP), consisting of cells that expressed numerous proliferation markers (Figure S13); eFibP cells were predominantly present at earlier time points, and their spatial distribution overlapped mainly with eFib3 (Figure 4C, Figure S12). These results demonstrate that endometrial fibroblast subtypes exhibit dynamic spatial organization.

Building on this spatial framework, we investigated how decidual cells emerge and organize during menstruation. Upon induction, two clusters, dec1 and dec2, emerged as concentric rings. At subsequent time points, a third cluster, dec3, arose at the center of these rings. This concentric organization persisted throughout later stages, with the central dec3 cluster expanding and the overall decidual tissue growing radially (Figure 4C, Figure S12). Notably, the concentric patterning of decidual clusters did not correlate with any change in expression of GsD, which we confirmed was present across all fibroblasts sub-populations (Figure S14). The final decidual cluster, decAp, was predominantly present in the menstrual sample and expressed many stress- and cell death–related genes, including the pro-apoptotic gene Bax, suggesting this population comprises dying cells as the tissue is shed (Figure S13).

The spatiotemporal organization of the decidual clusters suggested a model in which decidualization began near the luminal epithelium and the three decidual clusters represented cell states along a maturation gradient. In this model, the outermost dec1 cluster comprised the youngest, early-stage decidual cells; the intermediate dec2 cluster contained proliferating decidual cells; and the innermost dec3 cluster was composed of the most mature cells, which had initiated decidualization earliest and ultimately undergo cell death as menstruation initiated. A gradual increase in the expression of most decidual markers between dec1, dec2 and dec3 further supported this hypothesis (Figure S13).

To test this model, we performed pseudotime analysis on the final two timepoints, when the tissue was predominantly composed of decidual cells. Spatial mapping revealed a strong correlation between pseudotime values and radial position, with pseudotime values increasing from the periphery toward the center of the tissue (Figure 4D). This spatial pattern provided further evidence that the distinct decidual clusters represented a maturation gradient. Together, these data reveal previously unappreciated endometrial layering beyond the classical bilayered functionalis-basalis distinction. This layering arises as a consequence of a radial pattern of decidual cell differentiation that expands and compresses the outer layer of endometrial fibroblasts, creating a cleavage plane at the boundary between decidual and non-decidual cells.

## Discussion

We used chemogenetics to develop the first transgenic mouse model of menstruation, which recapitulates the core anatomical, functional, and transcriptional hallmarks of human menstruation. Our findings reveal previously unappreciated aspect of endometrial regulation, expanding our understanding of the classical functionalis-basalis dichotomy. Our robust, tractable in vivo model of menstruation offers new opportunities to dissect menstrual mechanisms within the complex endometrial environment and to understand communication between the menstruating uterus and the rest of the body, advancing our understanding of physiological and pathological processes associated with menstruation.

## Materials and methods

All material used in this study listed in Table S1.

### Animal husbandry.

All mice were housed under a 14:10 hour light–dark cycle at 21–25 °C and 30–70% humidity, with ad libitum access to food (irradiated LabDiet Prolab Isopro RMH 3000 5P75; LabDiet, St. Louis, MO) and water. Up to five animals were group-housed per cage (7.75” W × 12” L × 6.5” H) with coarse aspen Sani-chip bedding (PJ Murphy, Ladysmith, WI). All procedures were approved by the Institutional Animal Care and Use Committee of Harvard University and conducted in accordance with relevant ethical regulations and guidelines. Mouse strains used in this study are listed in Table S2.

### Induction of decidualization and menstruation in X-Mens mice.

Induction of pseudopregnancy: Female mice were paired with vasectomized C57BL/6 (Jax) or CD-1 (Charles River) males at a ratio of one or two females per male after 4 PM. Copulatory plugs were checked the following morning before noon. The day a plug was detected was designated as day 0.5 of pseudopregnancy, after which plugged females were separated from males.

On the evening of pseudopregnancy day 3.5 (after 4:00 PM), mice received 48 μg/mL clozapine-N-oxide dihydrochloride (CNO) in their drinking water. On pseudopregnancy day 4.5, animals were administered five consecutive intraperitoneal injections of a DREADD agonist cocktail (24 μg/mL CNO, 125 μg/mL deschloroclozapine dihydrochloride, and 200 μg/mL compound 21 dihydrochloride in physiological saline) at two-hour intervals. The following morning (pseudopregnancy day 5.5), CNO-supplemented water was replaced with regular drinking water prior to noon. Control animals received plain drinking water without CNO and were administered intraperitoneal injections of physiological saline on the same schedule as experimental animals. For the 1 dpi early timepoint, the animal was not given CNO in the drinking water and was induced only with intraperitoneal injections.

Assessment of menstruation: menstruation was monitored by collecting vaginal lavage samples beginning the day after DREADD agonist injections. Animals were gently restrained, and 30–100 μL of physiological saline was pipetted into the vagina and aspirated 5–10 times. A few microliters of the lavage sample were tested for the presence of red blood cells using using a fecal occult blood test (Hemoccult; HemoCue 60151A). The first day blood was detected in the lavage was designated as bleeding day 0.5. Daily lavages continued until blood was no longer detected or the uterus was collected. For non-menstruating animals, including controls, vaginal lavages were continued daily through pseudopregnancy day 11.5.

Repeated menstruation: cycles of pseudopregnancy, decidualization induction, and menstruation assessment were repeated as described. Animals were re-paired with vasectomized males to induce new cycles only after vaginal bleeding was absent for at least two consecutive days.

Mifepristone injection: on the evening of pseudopregnancy day 6.5 (after 4 PM), animals received a subcutaneous injection of 20 mg/kg mifepristone dissolved in 5% ethanol and 95% peanut oil. Following mifepristone administration, menstruation was monitored daily by vaginal lavage as described above. Animals that were already exhibiting vaginal bleeding at the time of mifepristone injection were excluded from analysis.

### Assessment of fertility following cessation of menstruation.

Post-menstrual X-Mens females were paired with intact, proven-breeder C57BL/6 males after 4:00 PM on the second consecutive day when no blood was detected in their vaginal lavages. Control animals, which received physiological saline instead of agonist during pseudopregnancy, were paired at pseudopregnancy day 11.5. Mating was confirmed by the presence of a vaginal plug the following morning, which was designated as gestational day 0.5. Plugged females were then separated from the males for the remainder of gestation.

GqD X-Mens females were monitored daily for parturition, and the number of pups born was counted within the first two days postpartum. GsD X-Mens females were euthanized by cervical dislocation between 10:00 AM and 1:00 PM on gestational day 9.5. Uteri were harvested and fixed overnight at 4 °C in 4% formaldehyde in PBS with gentle agitation. Samples were then washed three times in PBS, with each wash lasting between 15 minutes and 2 hours at room temperature, with gentle agitation. Implantation sites were quantified manually using an Olympus BX41 light microscope equipped with an Olympus UPLAN 4x objective lens.

### Organoid culture from menstrual effluent.

Sample collection: menstrual effluent was collected from X-Mens animals that underwent induction as described above and received mifepristone injections to synchronize bleedings (fig. S2A). Organoids were generated from pooled uterine lavage samples collected from pairs of females with copulatory plugs on the same day receiving identical treatments. Uterine lavages were collected at two time points from each animal: (1) pseudopregnancy day 3.5 (prior to agonist administration) and (2) pseudopregnancy day 7.5 (the day after mifepristone administration). For each time point, each mouse was lavaged with 100 μl of sterile saline; lavage fluids from each pair were pooled in transport medium consisting of Advanced DMEM, 10% fetal bovine serum, 10 mM HEPES, and 0.1 mg/mL primocin, and transported on ice.

Establishing organoid culture: cells were pelleted by centrifugation at 228 x g for 5 min at 4 °C. The pellet was resuspended in 150–250 μl of Matrigel, and 50 μl droplets were plated into individual wells of a 24-well plate. Plates were incubated at 37 °C for 10 min to solidify the Matrigel, after which 750 μl warm endometrial organoid medium (Advanced DMEM/F12 supplemented with 0.05 μg/mL human EGF, 0.1 μg/mL human Noggin, 0.1 μg/mL recombinant human or mouse R-spondin, 0.1 μg/mL human FGF, 0.05 μg/mL human HGF, 1:100 N2 supplement, 1:50 B27 supplement minus vitamin A, 0.1 mg/mL Primocin, 1 μM nicotinamide, 2 mM glutamine, 0.5 μM A83–01, 1.25 mM N-acetylcysteine, and 10 μM Y27632), was gently added to each well. Plates were then incubated at 37 °C, 5% CO_2_.

Organoid maintenance: organoids were maintained at 37 °C in a 5% CO_2_ humidified incubator. Media were replaced every 5 days by removing spent medium and adding 750 μl of fresh media. Organoid medium was prepared in bulk and stored at 4 °C; Y27632 was added freshly immediately before medium changes. The number of organoids per sample was counted manually between days 10–14 after initial plating. Subsequently, media were refreshed every 1–7 days, and organoids were passaged as required.

Organoid passaging: cultures were harvested by gently washing Matrigel droplets in PBS and releasing organoids with TrypLE Express. The mixture was incubated for 5–10 min at 37 °C and mechanically dissociated by pipetting with P1000 and P200 tips, shearing against the well bottom. Organoid fragments were transferred to 10 ml ADMEM plus 10% FBS and centrifuged at 228 x g for 5 min. The pellet was resuspended in Matrigel and replated in 50 μl droplets in a 24-well plate (1 droplet per well). The plate was incubated at 37 °C for 10 min; 500–750 μl of fresh endometrial organoid medium was added to each well. Standard passaging expanded one Matrigel droplet to four new droplets.

Organoid staining: matrigel-embedded org anoids were released using 1 ml Cell Recovery Medium, incubated on ice for 20 min, and washed with 9 ml ADMEM by centrifugation at 228 x g. Pellets were resuspended in a 1:1 mix of endometrial organoid medium and Matrigel and plated as 50 μl droplets into 96-well glass-bottom plates (Matriplate, Brooks). Plates were incubated at 37 °C for 10 min, then 500–750 μl endometrial organoid medium was added. After overnight incubation, organoids were fixed in 4% paraformaldehyde in PBS for 1 h at room temperature, blocked with 3% BSA in PBS with 0.1% Triton X-100, and incubated with primary antibodies overnight at 4 °C. Secondary antibodies were applied for at least 2 h at room temperature. (For details of antibodies and imaging, see below.)

### Harvesting Uteri.

Mice were euthanized either by exposure to CO_2_ gas for 5 minutes or by cervical dislocation; for Slide-Tags and RNA extraction experiments, only cervical dislocation was used. Following euthanasia, the abdominal skin and muscle layers were incised, and the reproductive tract was exposed. The uteri were excised by cutting the ovarian fat pads, mesometrium, and cervix. The mesometrium and ovarian fat pads were trimmed as needed.

Excised uteri were processed in three ways:

1. Imaging and fixation: immediately after dissection, uteri were arranged on filter paper rounds placed inside a 10” × 10” LED photo box under uniform lighting and photographed for documentation. Following imaging, tissues were fixed overnight at 4 °C in 4% formaldehyde in PBS with gentle agitation. Samples were washed three times in PBS (each wash lasting 15 minutes to 2 hours at room temperature, with gentle agitation) and then stored in PBS containing 0.1% sodium azide at 4 °C with gentle agitation.
2. RNA extraction: after dissection, each uterine horn (between the oviduct and the uterine body) was placed individually into sterile 1.5 mL microcentrifuge tubes, and snap frozen in liquid nitrogen. Samples were stored at –80 °C until use.
3. Slide-tags experiments: uterine horns were flash frozen in optimal cutting temperature (OCT) compound and stored at –80 °C until use.

### Total RNA extraction from X-Mens uteri and quantitative PCR analysis.

Cryopreserved uterine horns were processed immediately without thawing by direct dissociation in 1 mL RLT buffer (Qiagen) using a gentleMACS dissociator (Miltenyi) with gentleMACS M tubes, following the manufacturer’s instructions. 800 μl of the resulting tissue lysate was then transferred to a Qiashredder column (Qiagen) and centrifuged at 17,000 g for 3 minutes at 4 °C to remove tissue debris. Total RNA was isolated from the cleared lysate using the Qiagen RNeasy Mini Kit according to the manufacturer’s protocol. RNA concentration was determined using a Nano-Drop spectrophotometer (Thermo Scientific), and RNA samples were stored at −80 °C until further use.

cDNA synthesis: up to 2 μg of total RNA from each sample was treated with ezDNase (Thermo Fisher) to remove genomic DNA contamination, per the manufacturer’s protocol. First-strand cDNA synthesis was performed using the SuperScript IV First-Strand Synthesis System (Thermo Fisher) with oligo(dT) primers, following the manufacturer’s instructions. Synthesized cDNA was stored at −20 °C prior to quantitative PCR analysis.

### Quantitative PCR (qPCR).

Reactions were prepared in a final volume of 20 μl containing 10 μl PerfeCTa SYBR Green FastMix (Quanta Bio), 0.5 μl cDNA, 1 μl primer mix (10 μM stock concentration of each primer), and 8.5 μl nuclease-free water. For each sample, qPCR was performed for three genes: two housekeeping genes (Actb and Gapdh) and the decidualization marker Prl8a2. Each gene was measured in triplicate as a technical control, and for each experimental condition, at least three independent uterine samples were analyzed as biological replicates. qPCR was performed on a QuantStudio 7 real-time PCR system (Thermo Fisher). Relative expression levels of Prl8a2 were calculated using the ∆∆Ct method, with normalization to the uninduced pseudopregnant day 4.5 samples. Primers used for quantitative PCR are listed in Table S3.

### Paraffin sectioning and Hematoxylin & Eosin (H&E) staining.

Following fixation and PBS washes, uteri were transferred to 70% ethanol and stored at 4 °C with gentle agitation for at least 24 hours. Tissue dehydration and clearing were performed through a graded ethanol series, followed by xylene and paraffin infiltration. Specifically, samples were placed sequentially in 95% ethanol for 45 minutes and then 30 minutes, 100% ethanol for 45 minutes and then 30 minutes, xylene for 45, 60, and 30 minutes, and finally three changes of paraffin for 60 minutes each. After processing, samples were embedded in paraffin molds and sectioned at 5 μm using a microtome. Sections were mounted onto positively charged glass slides for staining.

For staining, paraffin sections were first deparaffinized in three successive washes of Histoclear II, each lasting 5 minutes, followed by rehydration through decreasing concentrations of ethanol and water. Specifically, slides were washed twice in 100% ethanol for 1 minute each, then in 95% ethanol for 30 seconds, 50% ethanol for 30 seconds, and twice in water for 1 minute each. Slides were then stained with hematoxylin for 1 minute, rinsed three times in water for 30 seconds each, and treated with High-Def solution for 30 seconds. After a further three water washes of 20 seconds each, slides were placed in bluing reagent for 1 minute, rinsed again three times in water for 20 seconds each, washed in 95% ethanol for 30 seconds, stained with eosin for 45 seconds, dehydrated in three 1-minute changes of 100% ethanol, and cleared in three 1-minute washes of Histoclear II. Stained slides were temporarily stored in Histoclear II prior to mounting. Slides were mounted using approximately 100 μL Permount mounting medium and immediately coverslipped, then allowed to cure for at least 24 hours before imaging.

Slides were imaged using a Zeiss Axioscan.Z1 microscope equipped with a Plan Apochromat 20×/0.8 objective and VIS LED light source under brightfield illumination. Image curation (resizing, cropping, adjusting the white balance and saturation) were performed in Fiji (Schindelin et al., 2012) as needed.

### Cryo-sectioning, staining, and imaging.

The fixed tissues (see Harvesting Uteri, approach 1) were PBS-washed and incubated overnight in 30% sucrose in PBS at 4 °C for cryoprotection. Following equilibration, tissues were embedded in OCT compound and snap frozen; OCT blocks were stored at –80 °C until sectioning.

For Slide-tags experiments, sections were generated from uteri fresh-frozen in OCT (see Harvesting Uteri, approach 3). One section was used for nuclei isolation and sequencing; a consecutive section was used for immunofluorescence.

Prior to sectioning, OCT blocks were equilibrated within the cryostat chamber at –19 to –21 °C for at least 30 minutes. Tissues were then sectioned at a thickness of 10–30 μm with a Leica CM 1860 cryostat, and the resulting sections were mounted onto positively charged glass slides. Prepared slides were stored at –80 °C until further use.

Immunofluorescence staining: cryosections were brought to 4 °C for 10 minutes to equilibrate, in a humid chamber. Fresh frozen sections were fixed in 4% formaldehyde in PBS for 15 minutes at room temperature, then washed three times for 15 minutes each in PBS containing 0.1% Tween-20 (PBT). For all samples, non-specific binding was blocked for 30–60 minutes at room temperature using blocking buffer composed of PBS with 5% normal donkey serum, 0.5% bovine serum albumin (BSA), 0.1% Triton X-100, and 0.1% sodium deoxycholate. After blocking, slide perimeters were marked with a hydrophobic barrier pen, and primary antibodies diluted in blocking buffer were applied to the tissue sections (approximately 150–300 μl per slide, depending on section size and the number of sections per slide). Slides were incubated with primary antibody either overnight at 4 °C or for 2 hours at room temperature in a humid chamber. After incubation, slides were washed three times for 15 minutes each in PBT. Secondary antibodies, also diluted in blocking buffer, were then applied at the same volume as the primaries, and slides were incubated either overnight at 4 °C or for 2 hours at room temperature in a humid chamber. DAPI (1 μg/ml final concentration) was included in the secondary antibody solution to serve as a nuclear counterstain. After completion of the staining protocol, slides were washed three times for 15 minutes each in PBT. Slides were then mounted using ProLong Gold Antifade mounting medium, coverslipped, and allowed to cure for at least 24 hours at room temperature prior to imaging. Antibodies used in this study are listed in Table S4.

Imaging: slides were imaged using a Yokogawa CSU-W1 spinning disk confocal system mounted on an inverted Nikon Ti-2E microscope, equipped with a CFI60 Plan Apochromat Lambda 20×/0.75 NA objective lens. Acquired images were analyzed using ImageJ software.

### Slide-tags.

Slide-tags assays were performed largely as described in the original publication (Russell et al., 2024), with several modifications. Barcoded bead arrays were manufactured and sequenced as previously described, using the 3′ feature barcode capture sequence ((TAGS beads): 5′-TTT-PC-GTGACTGGAGTTCAGACGTGTGCTCTTCCGATCTJJJJJJJJTCTTCAGCGTTCCCGAGAJJJJJJJNNNNNNNVVGCTTTAAGGCCGGTCCTAGCAA-3′). In addition to custom-manufactured arrays, transferred Curio barcoded bead arrays (Curio Biosciences) were also used.

To minimize bead loss, beads were transferred from their original substrate to an acrylamide-coated glass coverslip. First, 25 mm square coverslips were thoroughly cleaned and incubated for 15 minutes at room temperature in a solution of 0.2% (v/v) bind silane (3-(trimethoxysilyl)propyl methacrylate) and 1% (v/v) acetic acid in ethanol. After treatment, coverslips were washed three times in 100% ethanol and allowed to dry completely.

A fresh acrylamide solution was prepared on ice consisting of 0.6% (v/v) tetramethylethylenediamine (TEMED), 0.06% (v/v) ammonium persulfate (APS), and 10% (v/v) Acrylamide/Bis-acrylamide (19:1) in water. Approximately 30 μL of this acrylamide solution was applied to the bind silane-treated side of each coverslip. The Curio barcoded array was then placed bead-side down onto the acrylamide solution, ensuring that no bubbles formed between the array and the coverslip. An untreated coverslip was immediately placed on top to generate a thin acrylamide layer. The assembly was incubated undisturbed for 45 minutes at room temperature to allow polymerization.

After incubation, assemblies were gently separated. Successful bead transfer was verified by visual inspection. Excess acrylamide at the coverslip edges was removed, and the coverslips were stored for at least 12 hours at room temperature to ensure the acrylamide layer was completely dry before further use.

Tissue samples were fresh frozen and then cryosectioned to 20 or 30 μm thickness on a Leica CM1950 cryostat at −18 to −21 °C. Excess OCT was removed from sections using biopsy punches (3332P/25, Integra) before mounting tissue directly onto the barcoded array surface, taking care to avoid tissue folds. Except for the uninduced GsD X-Mens sample (in which two consecutive, small sections were placed side-by-side on a single array), only one cross-section was placed per array (for details, see fig. S6A). Sections were gently melted onto the arrays by warming with a finger placed beneath the barcoded array, then immediately transferred to a glass slide resting on ice. Each tissue section was then covered with 6–10 μL dissociation buffer (82 mM sodium sulfate, 30 mM potassium sulfate, 10 mM glucose, 10 mM HEPES, 5 mM magnesium chloride).

Spatial barcode release was initiated by exposure of the barcoded bead array to ultraviolet (365 nm) light (0.42 mW mm^−2^, Thorlabs, M365LP1-C5, LEDD1B) for 1 minute, followed by incubation on ice for 14 minutes to facilitate diffusion of the spatial barcodes. The barcoded array was then transferred to a 12-well plate (Corning, 3512) on ice, to which 2 mL of extraction buffer (dissociation buffer supplemented with 1% Kollidon VA64, 1% Triton X-100, 0.01% BSA, and 666 U mL^−1^ RNase inhibitor) was carefully added away from the tissue “puck.” Tissue was mechanically dissociated using a 200 μL pipette tip, performing 15–20 triturations as assessed by visual inspection, and the process was repeated until complete removal of tissue from the array. Further mechanical dissociation was performed with a 1000 μL pipette tip using 60–100 triturations, also monitored by visual inspection.

The resulting nuclei suspension was carefully aspirated and transferred, and the well was rinsed twice with 1 mL wash buffer (82 mM sodium sulfate, 30 mM potassium sulfate, 10 mM glucose, 10 mM HEPES, 5 mM magnesium chloride, 50 μL RNase inhibitor), and the rinses were pooled with the nuclei suspension to a final volume of 20 mL. Nuclei were pelleted by centrifugation at 600 g for 10 minutes at 4 °C in a pre-cooled swinging bucket centrifuge. After centrifugation, the supernatant was carefully removed, leaving 1 mL, and the pellet was resuspended and filtered through a pre-cooled, pre-wetted 40 μm cell strainer (Corning, 431750). This suspension was further centrifuged at 300 g for 10 minutes at 4 °C, the supernatant was removed to leave a final volume of 50 μL, and the pellet was resuspended. Nuclei were counted manually using a disposable hemocytometer.

A total of 43.3 μL of counted nuclei was loaded into the 10x Genomics Chromium controller using the Chromium Next GEM Single Cell 3′ Kit v3.1 (10x Genomics, PN-1000268). Subsequent steps were performed using the Chromium Next GEM Single Cell 3′ Reagent Kits v3.1 (Dual Index) with Feature Barcode Technology for Cell Surface Protein (Cell Ranger CG000317) according to the manufacturer’s protocol, with minor modifications. Spatial barcode libraries were prepared according to procedures for cell-surface protein library preparations, employing seven PCR cycles during the index PCR step (step 4.1f). For experiments using Curio barcoded bead arrays, 1 μL of 0.329 μM spike-in primer (5′-GTGACTGGAGTTCAGACGT-3′) was included during cDNA amplification, and the Index TT plate was used for the index PCR step.

### Slide-tags sequencing.

Single-cell RNA-seq and spatial barcode libraries were sequenced using either an Illumina NextSeq 1000 instrument with a P2 100 Cycle Kit (Illumina, 20046811) or an Illumina NovaSeq X instrument with a 300 Cycle Kit (Illumina).

### Slide-tags data preprocessing.

All code for processing Slide-tags data is publicly available at: https://github.com/thechenlab/Slide-tags Binary base call (BCL) files were demultiplexed using 10x Genomics Cell Ranger mkfastq (version 8.0.1) (Zheng et al., 2017). Gene expression reads were mapped to a custom reference genome that included the sequences for the GsD and GqD transgenes. This reference was generated using Cell Ranger mkref (version 7.2.0) (Zheng et al., 2017), and gene expression matrices were produced using Cell Ranger count (version 7.2.0). The resulting gene expression matrices were subsequently processed using CellBender remove-background (version 0.2.2), with the --total-droplets-included parameter set to 50,000 to reduce contamination from ambient RNA.

Reads corresponding to spatial barcode libraries were processed with custom Julia scripts (version 1.10.3) (Bezanson et al., 2017). Puck coordinate files were either acquired from internal databases or obtained through the Curio Bioscience Bead Barcode Whitelist Retrieval tool (https://knowledgebase.curiobioscience.com/bioinformatics/tile-barcode/) and converted to comma-separated values format. The Julia scripts parsed raw FASTQ files and removed reads that contained an ‘N’ in the UMI sequence, did not have a detectable universal primer (UP) site (permitting one deletion or up to two mismatches), or had a spatial barcode that did not match the whitelist CSV (allowing one mismatch or deletion). The scripts produced a dictionary in which each entry was identified by a triplet consisting of cell barcode, UMI, and spatial barcode, with values corresponding to read counts.

These dictionaries were further processed in R (version 4.3.3) using custom R scripts. Reads were further filtered to exclude chimeric sequences, reads with low-quality spatial barcodes, and those that did not correspond to a called cell. The remaining reads were collapsed into UMI counts for each cell and spatial barcode, yielding a spatial barcode count matrix. To assign spatial positions to each nucleus, DBSCAN clustering was used to classify spatial barcodes as either signal or noise. Hyperparameters (ε and minPts) were automatically optimized to achieve maximal accuracy in cell placement. For cells with a single robust signal cluster, the centroid was calculated by weighting nUMI values, and this centroid was assigned as the spatial coordinate for the cell.

### Quality control.

Unless otherwise specified, all quality control, preprocessing, and embedding steps were performed using the Scanpy package (v1.10.4) (Wolf et al., 2018). Cells with fewer than 500 unique molecular identifiers (UMIs), as corrected by CellBender, were excluded from analysis. In line with recommendations from the Single-cell Best Practices Consortium (Heumos et al., 2023), additional filters were applied: cells with either a log-transformed total UMI count or number of detected genes deviating by more than three median absolute deviations (MADs) from the overall median were removed. Cells with a mitochondrial gene fraction exceeding 8% of total UMIs were also excluded from downstream analysis.

To identify and remove doublets, we used the DoubletDetection Python package (v4.2) (Gayoso and Shor, 2020), employing the BoostClassifier class. For each sample, synthetic doublets were generated and combined with real cells, with joint clustering performed using Leiden clustering (Traag et al., 2019) on a principal component analysis (PCA) embedding constructed from the top 3,000 highly variable genes (determined within the package). In each iteration, a hypergeometric test assessed whether clusters were significantly enriched for synthetic doublets, comparing the observed number of synthetic doublets per cluster to expectation. The natural logarithm of the resulting p-value was recorded for all cells. Cells residing in clusters with a p-value below 10^−7^ were labeled as doublets for that iteration. The doublet detection procedure was repeated for 40 iterations, and any cell called as a doublet in at least 50% of iterations (voter_thresh=0.5) was labeled as a doublet. The number of predicted doublets across iterations was visually inspected to confirm convergence; all detected doublets were removed prior to further analysis.

After initial quality control, a total of 22,141 cells were removed from the mouse datasets and 27,127 from the human datasets, retaining 68,146 mouse and 70,250 human cells in the initial quality-controlled AnnData objects.

### Dimensionality reduction and clustering.

Individual samples were concatenated into multi-sample AnnData objects, constructed separately for human and mouse datasets (Virshup et al., 2024). Dimensionality reduction was performed on each dataset using a scVI variational autoencoder implemented in the scvi-tools Python package (v1.1.2) (Lopez et al., 2018). For each dataset, scVI models were trained on the top 3,000 highly variable genes (HVGs), excluding mitochondrial and ribosomal genes and identified using scanpy.pp.highly_variable_genes() with flavor set to seurat_v3 on unnormalized count matrices. Sample labels representing technical replicates were used as the batch key. Unnormalized counts were provided as input to the autoencoder, which was configured with two encoder/decoder layers, 30 latent variables, and a negative binomial likelihood. Models were trained for the default number of epochs, monitoring the evidence lower bound (ELBO) to ensure convergence, and with early stopping enabled.

For each scVI latent embedding, a k-nearest neighbors graph (k = 100) was constructed. Leiden clustering was performed using scanpy.tl.leiden() with the igraph implementation (Csardi and Nepusz, 2006) and random_state=0 at resolutions from 0.1 to 1.0 in increments of 0.1, as well as 1.5, 2.0, 2.5, and 3.0. To assess the relationships between cluster assignments across resolutions, hierarchical visualizations were generated using the clustree algorithm (v0.5.1) (Zappia and Oshlack, 2018). From these visualizations, a Leiden resolution of 0.1 was chosen for downstream annotation of coarse cell types. Two-dimensional visualizations were generated using Uniform Manifold Approximation and Projection (UMAP) (McInnes et al., 2018), computed via scanpy.tl.umap() using the same nearest-neighbors graph, with a minimum distance (min_dist) parameter of 0.5 and spread parameter of 1.0.

### Data curation and cell type annotation.

Canonical marker genes defined in previously published mouse and human endometrial single-cell expression datasets (Kirkwood et al., 2021; Kirkwood et al., 2022; Wang et al., 2020; Winkler et al., 2024) were evaluated in each cluster using scanpy.pl.dotplot(). Clusters exhibiting similar marker gene expression profiles were merged and assigned a unified annotation, while clusters presenting mixed marker gene profiles were labeled as such and excluded from further downstream analysis.

Following annotation, lineage-specific sub-objects were re-integrated using previous scVI model parameters. Some cells re-clustered with alternate broad lineages after reintegration; these were identified by constructing a k-nearest neighbors graph (k = 100) in the UMAP embedding of the respective lineage subset, and cells with a mean neighborhood distance greater than three MADs above the median were excluded (518 cells in mouse, 692 cells in human). After cell type annotation and post-processing, the human AnnData object contained 69,525 cells and the mouse AnnData object contained 57,242 cells.

To support cell state annotation and identify marker genes, differential expression analysis was conducted using the scanpy.tl.rank_genes_groups() function, applying the Wilcoxon rank-sum test to log-normalized gene expression values. For this analysis, count matrices were first normalized to 10,000 unique molecular identifiers (UMIs) per cell with scanpy.pp.normalize_total() and then log-transformed using scanpy.pp.log1p(). Genes were compared between cell type annotations using the Wilcoxon rank-sum test as implemented in scanpy.tl.rank_genes_groups(). Resulting p-values were adjusted for multiple hypothesis testing using the Benjamini-Hochberg procedure (Benjamini and Hochberg, 1995).

Pseudotime inference was performed on endometrial fibroblasts and decidual cells from all mouse timepoints to reconstruct dynamic cellular trajectories. A joint, unintegrated scVI embedding with 30 latent dimensions was computed and used to construct a k-nearest neighbors graph (k = 15). From this graph, a diffusion map with 10 components was generated using scanpy.tl.diffmap(), and diffusion pseudotime was estimated with scanpy.tl.dpt() (Haghverdi et al., 2016). The root cell for pseudotime estimation was set as an endometrial fibroblast from the uninduced sample; results were robust to the specific fibroblast chosen as the root. All 10 diffusion map components were used in the computation of pseudotime.

### Pseudotime-associated genes.

To identify genes associated with pseudotime, we employed a mutual information-based approach adapted from previous studies on the menstrual cycle and mouse pregnancy atlases (Wang et al., 2020; Winkler et al., 2024). For mice, data from the early (1 dpi) and late (bleeding, 4 dpi) samples were concatenated into a common AnnData object. Highly variable gene selection was performed using the same criteria as described above, selecting the top 3,000 highly variable genes (HVGs) with sample labels specified as the batch key. An integrated scVI embedding was created, and diffusion pseudotime was inferred from a k-nearest neighbors graph (k = 15) constructed on this latent embedding. An outer endometrial fibroblast cell was specified as the root. An analogous procedure was carried out independently a joint embedding containing both human samples (menstrual and secretory).

For each expressed gene, we estimated differential mutual information between log-normalized expression counts and diffusion pseudotime values using the k-nearest neighbors estimator implemented in sklearn.feature_selection.mutual_info_regression() with default settings (n_neighbors = 3) (Kraskov et al., 2004). To estimate false discovery rates, we performed 1,000 permutations by shuffling pseudotime values using numpy.random.default_rng.shuffle(), re-estimating mutual information for each shuffle. For each gene, an empirical one-sided permutation p-value was computed as the proportion of permutations with mutual information equal to or exceeding the observed value. Resulting p-values were then adjusted for multiple hypothesis testing using the Benjamini-Hochberg procedure. Genes with a q-value below 0.05 were considered significant, resulting in 14,636 significant genes for the human dataset and 14,244 for the mouse dataset. To focus on the strongest pseudotime associations, we calculated the 90th percentile of mutual information scores among significant genes in each species; genes above this threshold were designated as top pseudotime-associated hits. (fig. S16).

To compare pseudotime-associated genes across species, orthology relationships between Mus musculus and Homo sapiens were obtained from the Mouse Genome Informatics (MGI) database (Baldarelli et al., 2024) (downloaded from https://informatics.jax.org/downloads/reports/HOM_MouseHumanSequence.rpt) and filtered to retain only strict one-to-one gene pairs. The background set for cross-species comparison included all one-to-one orthologs that were (i) expressed in at least 1% of fibroblasts and decidual cells in both species and (ii) identified as significant for mutual information in both. Overlap between the top mouse and top human gene sets was then assessed with a hypergeometric test using scipy.stats.hypergeom(), where N is the total number of background orthologs (9,731 genes), K is the number of top mouse genes with a strict one-to-one ortholog (1,182 genes), n is the number of top human genes with a strict one-to-one ortholog (970 genes), and k is the observed intersection (302 genes). This test evaluates whether the observed overlap exceeds the expectation under a null hypothesis of random overlap, with the expected value given by K × n / N (approximately 118 genes, p ≈3.2 × 10^−63^).

### Visualization of pseudotime-associated gene dynamics.

To visualize the dynamics of pseudotime-associated genes, we generated heatmaps of smoothed expression trajectories for the top pseudotime-associated mouse genes and their human orthologs. For each gene, log-normalized expression values were extracted from the relevant AnnData object and modeled as a smooth function of pseudotime using a generalized additive model (GAM) with a spline basis. Specifically, GAMs were fit using the LinearGAM implementation from the pyGAM package (v0.10.1) (Servén and Brummitt, 2018), employing 20 splines as the basis. Smoothed expression predictions were obtained on a uniform grid of 800 pseudotime points.

To facilitate cross-gene comparability and account for differences in dynamic range, predicted expression values were quantile-normalized: for each gene, the 1st percentile was subtracted from predicted values, and the result was divided by the 99th percentile, yielding normalized values ranging from 0 to 1. This normalization ensured that patterns in gene activation could be comparably visualized. Finally, genes were ordered in the heatmap according to the pseudotime value at which their predicted expression peaked, thereby highlighting temporal waves of gene activation.

### Cross-species integration with SAMap.

To enable cross-species mapping of endometrial fibroblasts and decidual cells via SAMap, we used Ensembl reference transcriptomes for sequence alignment (Homo sapiens GRCh38, release 113; Mus musculus GRCm39, release 113) (Harrison et al., 2024). Transcript version suffixes were removed from FASTA headers, and a single canonical transcript per gene was retained. Gene symbols in the AnnData objects were then mapped to canonical transcript identifiers; genes lacking an associated transcript ID were discarded from further analysis.

Separate AnnData objects containing human and mouse endometrial fibroblasts and decidual cells (one for each species) were prepared with canonical transcript IDs as gene indices (adata.var) and log-normalized counts were stored in the primary expression matrix (adata.X). These objects were provided as input to SAMap version 1.0.15 (Tarashansky et al., 2021), together with reciprocal tblastx similarity scores between the transcriptomes (Altschul et al., 1990; Camacho et al., 2009) (BLAST+ v2.9.0). SAMap was run in pairwise mode to integrate data from the two species and generate a joint embedding.

We performed SAMap integration for two cross-species stage pairs: (i) mouse 1 dpi (late) with the human secretory phase sample, and (ii) mouse 4 dpi (bleeding) with the human menstrual phase sample. For each SAMap joint embedding, a k-nearest neighbors graph (k = 15) was constructed. Leiden clustering was then performed using the igraph implementation in Scanpy, with a resolution of 0.2 for the 1 dpi–secretory embedding and 0.15 for the 4 dpi–menstrual embedding, which each produced 3 clusters. Cluster assignments were visualized spatially by overlaying Leiden clusters on Slide-tags spatial coordinates, with individual clusters highlighted by color.

### Spatial pseudotime (vector field visualization).

To visualize spatial patterns in pseudotime during mouse decidualization, we analyzed decidual cells and endometrial fibroblasts from the three- and four-day post-induction samples. For each sample, a separate scVI embedding was constructed using the same parameters as those used for the multi-sample mouse pseudotime analysis. The root cell was specified as an outer endometrial fibroblast from the uninduced region; results were robust to the specific cell chosen. Diffusion pseudotime was inferred as previously described, using the scVI embedding as input.

Prior to estimating the pseudotime gradient vector field, spatial outliers were removed. Outlier detection was performed by constructing a k-nearest neighbors graph (k = 5) in physical space using the Nearest-Neighbors implementation from scikit-learn (v1.4.2) (Pedregosa et al., 2011). For each cell, both the mean distance to its five closest neighbors and the mean absolute pseudotime difference relative to these neighbors were computed. Cells with a spatial distance above the 98th percentile and a pseudotime difference above the 95th percentile, calculated using numpy.quantile() (numpy v1.26.4), were classified as outliers and excluded from further analysis (Harris et al., 2020).

For spatial visualization, each cell was colored according to its inferred pseudotime value and plotted in its physical Slide-tags (x, y) coordinates (Russell et al., 2024). To estimate a continuous pseudotime field, a Gaussian process regression (GPR) was fit to the filtered pseudotime values using sklearn.gaussian_process.GaussianProcessRegressor, using a kernel defined as the sum of a radial basis function (RBF, initial length scale = 1.0) and a white noise kernel (initial variance = 10–3) (Rasmussen and Williams, 2019). The fitted GPR provided a smooth mean function for pseudotime over the spatial domain. This function was evaluated on a regular 200 × 200 grid spanning the tissue section, and local spatial gradients of this mean pseudotime function were computed directly on the grid using central finite differences implemented in numpy.gradient(). To reduce high-frequency noise introduced by interpolation and by bias in spatial mapping, the pseudotime suface was convolved with a Gaussian blur (σ = 10 grid pixels; scipy.ndimage.gaussian_filter(); SciPy v1.12.0) (Virtanen et al., 2020). Note that Gaussian blur may smooth out meaningful local anisotropies, and particularly for the 4dpi sample. These gradient streamlines are primarily for visualization of the radial variation of pseudotime values. The convex hull of observed spatial coordinates was computed (scipy.spatial.ConvexHull) (Barber et al., 1996), and grid points outside the hull were masked. The resulting gradient vector field was visualized as streamlines using matplotlib.pyplot.streamplot(), with each streamline reflecting the direction and magnitude of the smoothed two-dimensional spatial gradient of pseudotime across the tissue section.

## Supplementary Material

Supplement 1

Supplement 2

Supplement 3

## Data Availability

Requests for materials should be directed to the corresponding author: kara_mckinley@harvard.edu. Code is available at github.com/McKinleyLab. Mouse and human sequencing data are available upon request, and will be released publicly upon publication.
