# Supplementary material for: Induction of menstruation in mice reveals the regulation of menstrual shedding
Source: bioRxiv. 2025 Oct 9:2025.10.08.681007. Preprint. [Version 1] doi: 10.1101/2025.10.08.681007 (PMC12668411; doi:10.1101/2025.10.08.681007)
Supplement: Supplement 2 [file media-2.pdf]

Induction of menstruation in mice reveals the regulation of menstrual shedding

Çağrı Çevrim[1], Nicholas J. Hilgert[2], Aellah M. Kaage[1], Andrew J.C. Russell[1,3], Allison E. Goldstein[1,4], Claire J. Ang[1,2], Jaina L.R. Gable[1,5], Laura E. Bagamery[6], Ana Breznik[1,5], Daniela J. Di Bella[1], Mustafa Talay[2,5] Jingyu Peng[1,4], Kathleen E. O'Neill[7], Fei Chen[1,3], Sean R. Eddy[2,5], Kara L. McKinley[1,5]*

1 Department of Stem Cell and Regenerative Biology, Harvard University, Cambridge MA, 02138, USA
2 Department of Molecular and Cellular Biology, Harvard University, Cambridge MA, 02138, USA
3 Broad Institute, Cambridge MA 02142, USA
4 Biological and Biomedical Sciences Program, Harvard University, Cambridge MA, 02138, USA
5 Howard Hughes Medical Institute, Cambridge MA 02138, USA
6 Department of Systems Biology, Harvard University, Boston MA 02115, USA
7 Department of Obstetrics and Gynecology, University of Pennsylvania, Philadelphia PA 19104
* *Author for correspondence (kara_mckinley@harvard.edu)*

Supplementary materials

This file includes:
- Supplementary Text
- Figures S1 to S16

Other Supplementary Materials include:
- Data S1

Supplementary Text

Transgenic strains for menstruation induction. To express DREADDs in the endometrium, we employed the Cre/Lox binary expression system (Orban et al., 1992). To elevate cAMP, we combined the Cre-inducible GsD line (Akhmedov et al., 2017), together with the Amhr2-Cre line (Jamin et al., 2002). The expression pattern of *Amhr2* and the resulting recombination induced by this Cre line have been subject to ongoing debate (Dickson et al., 2023; Huang et al., 2012; Patterson et al., 2013; Spooner-Harris et al., 2023). In our experiments, we consistently observed recombination in the endometrial fibroblasts, as well as myometrium and, only occasionally, the endometrial epithelium (Figure S1). We used $Amhr2^{Cre/+}R26^{GsD/+}$ and $Amhr2^{Cre/+}R26^{GsD/GsD}$ genotypes for our studies.

For the second strain, we combined a Cre-inducible GqD (Zhu et al., 2016) with the *Ltf-iCre* line (Daikoku et al., 2014). *Ltf-iCre* is reported to be active in the endometrial epithelium and in neutrophils, which we also confirmed in our analysis (Figure S1). Only $Ltf^{iCre/+}Tg^{GqD/0}$ animals were used in these experiments.

Induction of decidualization in X-Mens mice. Decidualization was induced using a combination of water-soluble clozapine N-oxide (CNO) delivered in drinking water and five consecutive intraperitoneal injections of a mixture containing three DREADD agonists (water-soluble forms of CNO, deschloroclozapine, and compound 21). Although not systematically tested, we observed that using only drinking water or only intraperitoneal delivery, or reducing the number of injections, led to decreased bleeding efficiency.

Pregnancy success in X-Mens mice following menstruation. A key hallmark of human menstruation is the ability of the endometrium to regenerate after shedding and support subsequent pregnancies. To assess endometrial function after induced menstruation, we evaluated fertility outcomes in X-Mens mice. GqD X-Mens mice exhibited equivalent litter sizes in non-menstruated and menstruated groups, indicating that induced menstruation did not affect fertility (Figure S4). Non-menstruated (control) GsD X-Mens females experienced late-gestation miscarriages, so fertility in this strain was assessed only through early pregnancy outcomes. Quantification of implantation sites on gestational day 9.5 showed comparable numbers between menstruated and control animals, indicating that menstruation does not impede subsequent embryo implantation.

Comparison of GqD and GsD X-Mens strains. There are key biological and practical differences between GsD and GqD X-Mens mice. In GsD X-Mens, all fibroblasts receive the decidualization signal systemically,

paralleling human physiology, while in GqD X-Mens, the signal is transduced through the epithelium, more closely mirroring the mechanism during mouse pregnancy. Practically, GsD X-Mens mice are infertile regardless of exposure to agonist, potentially due to basal activity of the GsD receptor in the absence of agonist. Although we use a GFP-fused GsD with lower basal activation than the original GsD, activation is not completely absent (Nakajima et al., 2016). Additionally, GsD X-Mens mice tend to develop vaginal prolapse after multiple rounds of induced menstruation; we observed prolapse in (n=11/14) of mice after repeated induction. Similar prolapse occurred in animals repeatedly given agonist without pseudopregnancy (n=2/4), suggesting the phenotype may relate to Amhr2-Cre activity outside the uterus.

In contrast, GqD X-Mens mice do not exhibit infertility or develop prolapse, but display lower bleeding efficiency (Figure 1E), potentially due to epithelial mosaicism of the Ltf-iCre line (not directly tested).

Future development of alternative approaches, using different Cre lines, AAV-mediated DREADD delivery, or engineering improved DREADD proteins may further enhance the versatility and precision of this method.

Staging of samples and annotation of single cell clusters. For GsD X-Mens samples, we collected a time course starting from the day after induction and confirmed the progression of decidualization by Cdh3 immunostaining. To verify that the samples represented a decidualization time course, we performed pseudotime analysis of all fibroblasts and decidual cells from all samples (without integration). These analyses confirmed that cells from each time point were ordered as expected based on Cdh3 staining (Figure S15).

Menstrual cycle staging of human samples was initially performed by examination of H&E sections by a gynecologic pathologist. The menstrual sample was confidently assigned, while the other sample was designated as secretory or inactive; we confirmed the secretory stage via Slide-tags data based on the presence of decidual cells.

Major cell lineages were annotated using established marker genes. For the mouse dataset, epithelial cells were identified using *Cdh1* and *Pax8*; endometrial fibroblasts with *Pdgfra*; decidual cells with *Wnt4*, *Prl8a2*, *Bmp2*, and *Cdh3*; vasculature cells with *Flt1* and *Pecam1*; immune cells with *Ptprc*; and smooth muscle cells with *Acta2* and *Myh11*. For the human dataset, epithelial cells were identified with *EPCAM* and *KLF5*; endometrial fibroblasts with *COL5A1* and *COL6A3*; decidual cells with *LEFTY2* and *IGFBP1*; immune cells with *PTPRC* and *STK17B*; vasculature cells with *PECAM1* and *VWF*; and smooth muscle cells with *ACTA2* and *MYH11* (Figure S6). Low-resolution Leiden clustering (resolution 0.1) robustly separated the main cell types in both human and mouse datasets. Notably, in mice, we discovered a previously unrecognized *Sox6*-expressing fibroblast-like cluster at the outermost layer of the uterine wall, likely corresponding to perimetrium cells (Figure S6).

Identification of endometrial fibroblast and decidual cell subtypes. We generated a combined annotated data (AnnData) object containing only the count vectors for fibroblast and decidual cells from all X-Mens samples (anndata v0.10.8). Leiden clustering was performed at a resolution of 0.8 to identify subclusters; 0.8 was chosen to ensure robust cross-sample identification as higher resolutions resulted in sample-specific cluster separation (Figure S15A). Among the four endometrial fibroblast clusters identified in the uninduced endometrium, eFib2 was almost absent after induction. Its spatial position was instead occupied by a distinct fibroblast cluster. Analysis using the clustree package (Zappia and Oshlack, 2018) (Figure S13D) revealed high similarity between these two clusters, suggesting that induction alters the transcriptome of eFib2 cells, so they form a separate cluster at the selected Leiden resolution. We therefore named this new cluster eFib2-induced (eFib2i; Figure S13, B and C).

The basal eFib1 fibroblast cluster remains localized to the basalis throughout decidualization and menstruation, and these cells do not contact decidual populations (Figure S12), suggesting they do not undergo decidualization. This finding is consistent with previous reports of non-decidualizing basal fibroblasts in mouse endometrium (Kirkwood et al., 2022). Notably, eFib1 cells also express very low levels of the progesterone receptor gene (*Pgr*), which may explain their resistance to decidualization (Figure S13B). The subluminal eFib3 population diminishes as decidualization progresses, indicating that these are the primary cells undergoing decidualization. Supporting this, the eFibP proliferating fibroblast population spatially overlaps with eFib3 (Figure S12), consistent with the known involvement of cell proliferation in early decidualization (Ramathal et al., 2010). However, we do not exclude the possibility that eFib2 may also contribute to the decidua.

Among the three decidual subtypes identified, the intermediate dec2 subtype expresses proliferation markers such as *Top2a*, *Kif15*, and *Mki67*, suggesting proliferative activity in this population (Figure S15B).

Cross-species comparison of pseudotime-associated genes. To identify pseudotime-associated genes in each species, we focused on genes whose expression patterns were most informative about the progression of a cell along the inferred pseudotime trajectory (Wang et al., 2020; Winkler et al., 2024). We quantified this informativeness by measuring how much the expression of a gene reduced uncertainty about the position of a cell in pseudotime, and assessed the statistical significance against a randomized background. Genes whose associations exceeded this expectation were considered pseudotime-associated. To focus on the most relevant genes, we highlighted those with the strongest associa-

tions in each dataset (see Methods and Figure S16 for details).

To compare pseudotime-associated genes between mouse and human, we first identified one-to-one gene pairs (orthologs) shared by the two species. Only ortholog pairs for which both genes were expressed and significantly associated with pseudotime in both datasets were included in the analysis. We then quantified how many of the top pseudotime-associated genes in mouse had matching, top-ranked orthologs in human. To determine whether this overlap was greater than expected by chance, we used a hypergeometric test, a standard statistical test that takes into account the total number of orthologous genes considered, the number of top-associated genes in each species, and the observed number of shared genes (see Methods for details). It is important to note that this approach may underestimate the true extent of overlap, since some genes, such as the prolactin gene family, have more complex and non–one-to-one orthology relationships. Nevertheless, our analysis revealed that the observed concordance between mouse and human pseudotime-associated gene signatures was significantly greater than would be expected by random overlap.

References

Akhmedov, D., Mendoza-Rodriguez, M. G., Rajendran, K., Rossi, M., Wess, J. and Berdeaux, R. (2017). Gs-DREADD Knock-In Mice for Tissue-Specific, Temporal Stimulation of Cyclic AMP Signaling. *Mol. Cell. Biol.* 37.

Daikoku, T., Ogawa, Y., Terakawa, J., Ogawa, A., DeFalco, T. and Dey, S. K. (2014). Lactoferrin-iCre: a new mouse line to study uterine epithelial gene function. *Endocrinology* 155, 2718–2724.

Dickson, M. J., Gruzdev, A. and DeMayo, F. J. (2023). iCre recombinase expressed in the anti-Müllerian hormone receptor 2 gene causes global genetic modification in the mouse. *Biol. Reprod.* 108, 575–583.

Huang, C.-C., Orvis, G. D., Wang, Y. and Behringer, R. R. (2012). Stromal-to-epithelial transition during postpartum endometrial regeneration. *PLoS One* 7, e44285.

Jamin, S. P., Arango, N. A., Mishina, Y., Hanks, M. C. and Behringer, R. R. (2002). Requirement of Bmpr1a for Müllerian duct regression during male sexual development. *Nat. Genet.* 32, 408–410.

Kirkwood, P. M., Gibson, D. A., Shaw, I., Dobie, R., Kelepouri, O., Henderson, N. C. and Saunders, P. T. K. (2022). Single-cell RNA sequencing and lineage tracing confirm mesenchyme to epithelial transformation (MET) contributes to repair of the endometrium at menstruation. Elife 11.

Nakajima, K.-I., Cui, Z., Li, C., Meister, J., Cui, Y., Fu, O., Smith, A. S., Jain, S., Lowell, B. B., Krashes, M. J., et al. (2016). Gs-coupled GPCR signalling in AgRP neurons triggers sustained increase in food intake. *Nat. Commun.* 7, 10268.

Orban, P. C., Chui, D. and Marth, J. D. (1992). Tissue- and site-specific DNA recombination in transgenic mice. *Proc. Natl. Acad. Sci. U. S. A.* 89, 6861–6865.

Patterson, A. L., Zhang, L., Arango, N. A., Teixeira, J. and Pru, J. K. (2013). Mesenchymal-to-epithelial transition contributes to endometrial regeneration following natural and artificial decidualization. *Stem Cells Dev.* 22, 964–974.

Ramathal, C. Y., Bagchi, I. C., Taylor, R. N. and Bagchi, M. K. (2010). Endometrial decidualization: of mice and men. *Semin. Reprod. Med.* 28, 17–26.

Spooner-Harris, M., Kerns, K., Zigo, M., Sutovsky, P., Balboula, A. and Patterson, A. L. (2023). A re-appraisal of mesenchymal-epithelial transition (MET) in endometrial epithelial remodeling. *Cell Tissue Res.* 391, 393–408.

Wang, W., Vilella, F., Alama, P., Moreno, I., Mignardi, M., Isakova, A., Pan, W., Simon, C. and Quake, S. R. (2020). Single-cell transcriptomic atlas of the human endometrium during the menstrual cycle. *Nat. Med.* 26, 1644–1653.

Winkler, I., Tolkachov, A., Lammers, F., Lacour, P., Daugelaite, K., Schneider, N., Koch, M.-L., Panten, J., Grünschläger, F., Poth, T., et al. (2024). The cycling and aging mouse female reproductive tract at single-cell resolution. *Cell* 187, 981-998.e25.

Zappia, L. and Oshlack, A. (2018). Clustering trees: a visualization for evaluating clusterings at multiple resolutions. *Gigascience* 7.

Zhu, H., Aryal, D. K., Olsen, R. H. J., Urban, D. J., Swearingen, A., Forbes, S., Roth, B. L. and Hochgeschwender, U. (2016). Cre-dependent DREADD (designer receptors exclusively activated by designer drugs) mice. *Genesis* 54, 439–446.

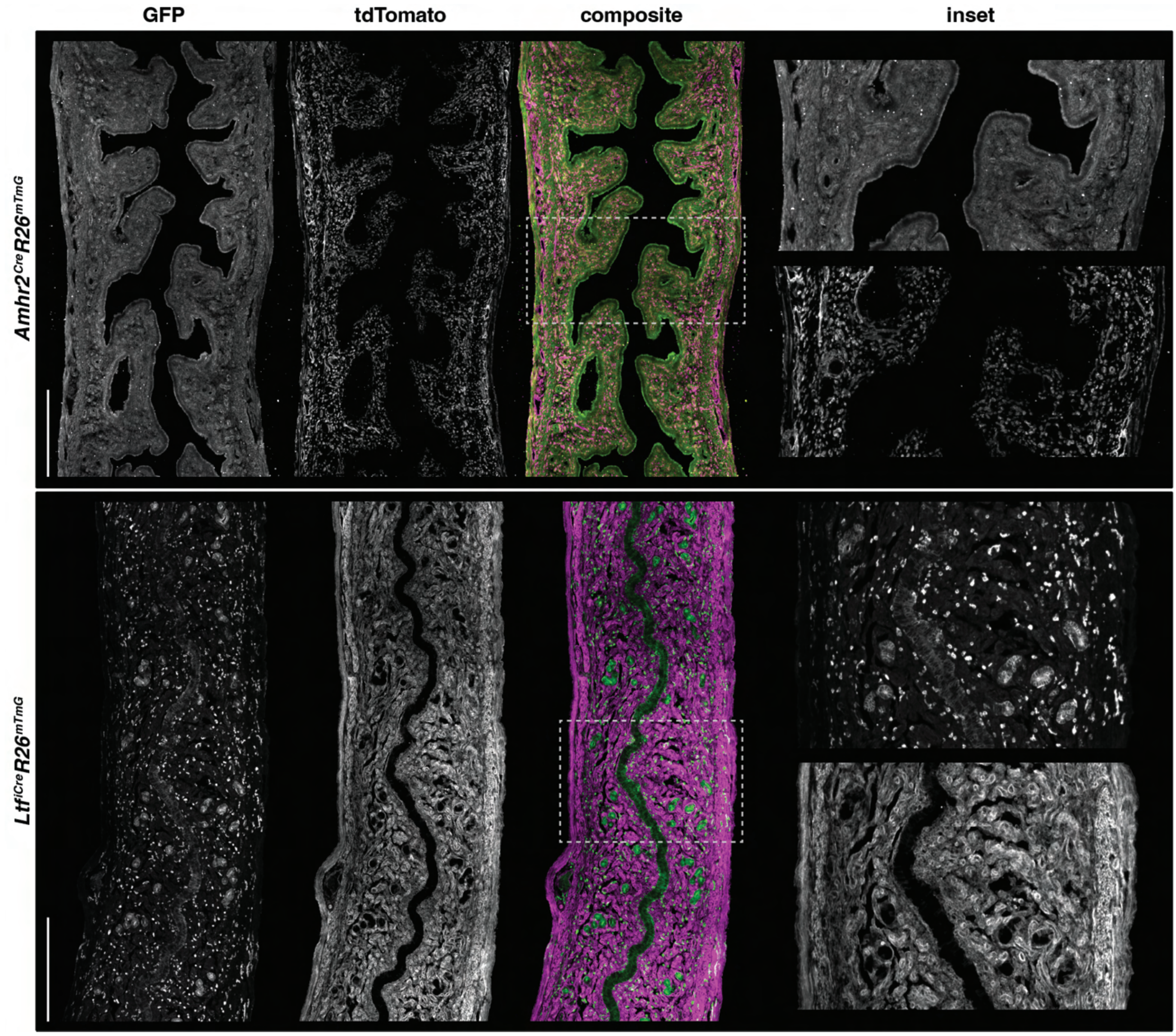

Figure S1. Lineage tracing of Cre lines used for the X-Mens method.
Immunofluorescence images of uterine longitudinal sections from Amhr2-Cre (top) and Ltf-iCre (bottom) mice crossed with R26mTmG reporter mice. Cre-positive cells are labeled with membrane-bound GFP and Cre-negative cells express membrane-bound tdTomato. Composite images show the merged channels. Scale bars: 500 μm.

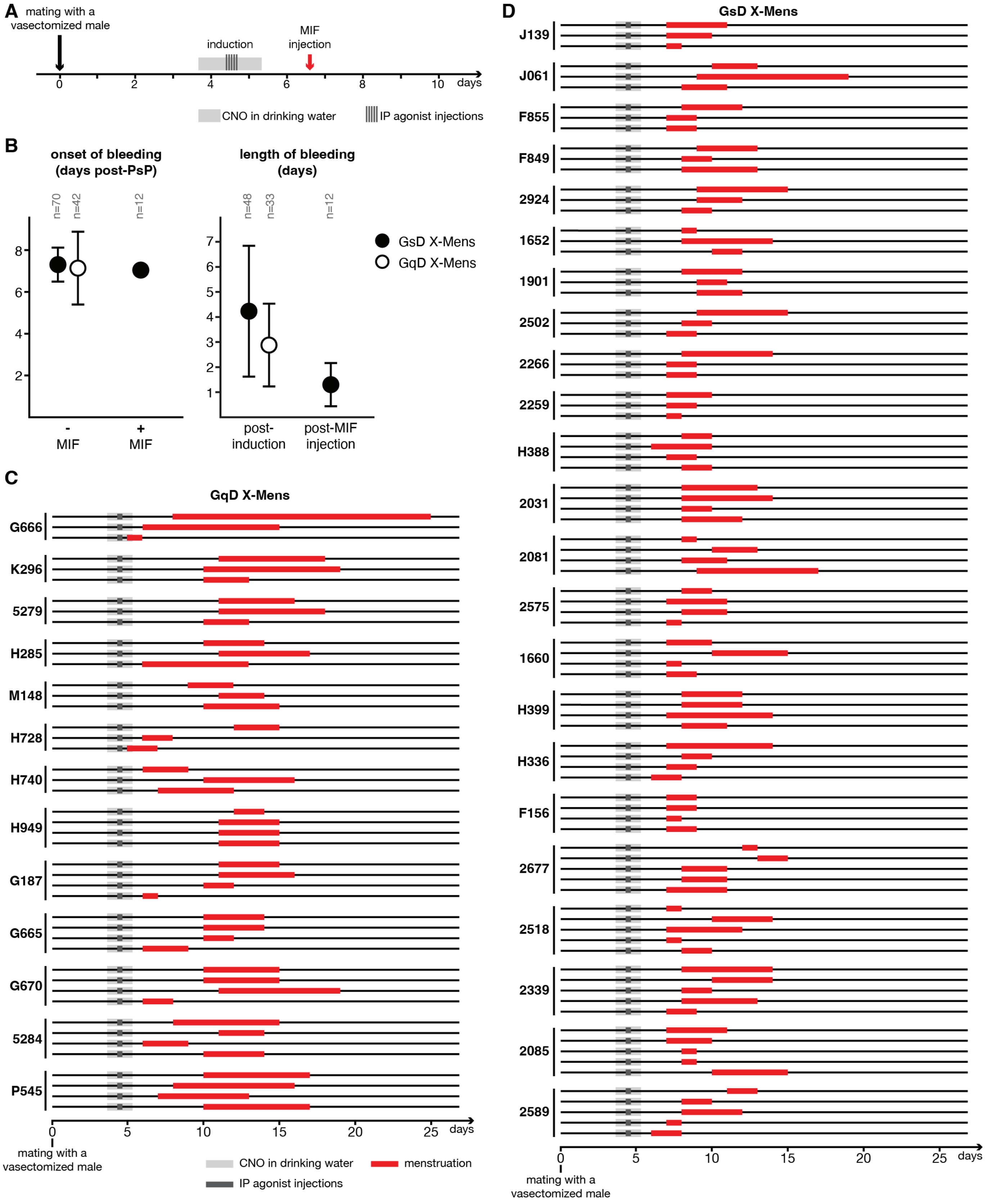

Figure S2. Induction protocol and variability in onset and duration of bleeding in X-Mens models.
A. Schematic of the induction protocol with optional Mifepristone (MIF) injection step. **B.** Onset (left) and length (right) of bleeding after induction, with and without MIF injection, in GsD and GqD X-Mens mice (mean ± SD). PsP: pseudopregnancy. **C.** Individual bleeding episodes for each GqD X-Mens mouse, and **D.** each GsD X-Mens mouse, all of which underwent repeated menstruation. Animal ID is shown on the left and the bottom row indicates the first menstruation; the top row is the last menstruation.

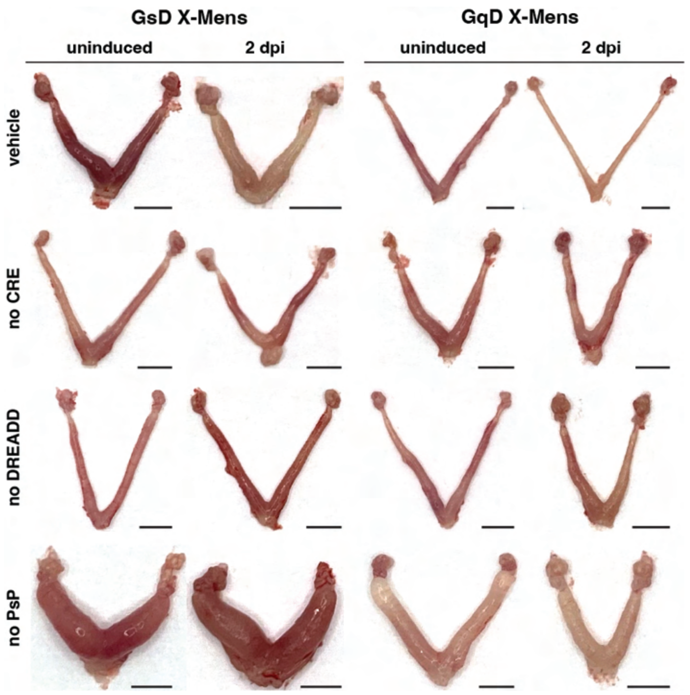

Figure S3. Representative images of X-Mens uteri from experimental controls.
Top row: animals receiving vehicle only; second row: genetic controls lacking the Cre allele; third row: genetic controls lacking the DREADD allele; bottom row: animals carrying both alleles induced with agonist but without pseudopregnancy (PsP). Scale bars: 5 mm.

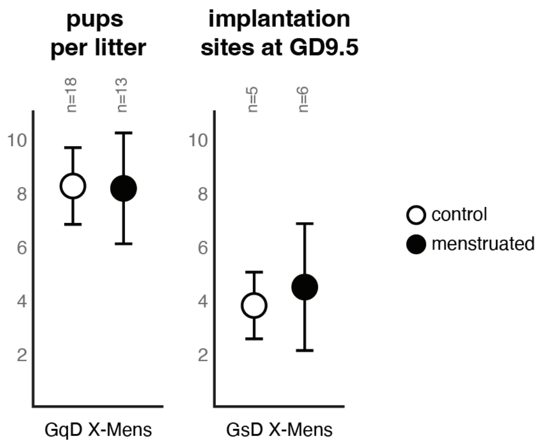

Figure S4. Pregnancy outcomes in X-Mens mice.
The number of pups per litter in menstruated and control GqD X-Mens mice (left), and the number of implantation sites at gestational day 9.5 (GD9.5) in menstruated and control GsD X-Mens mice (right).

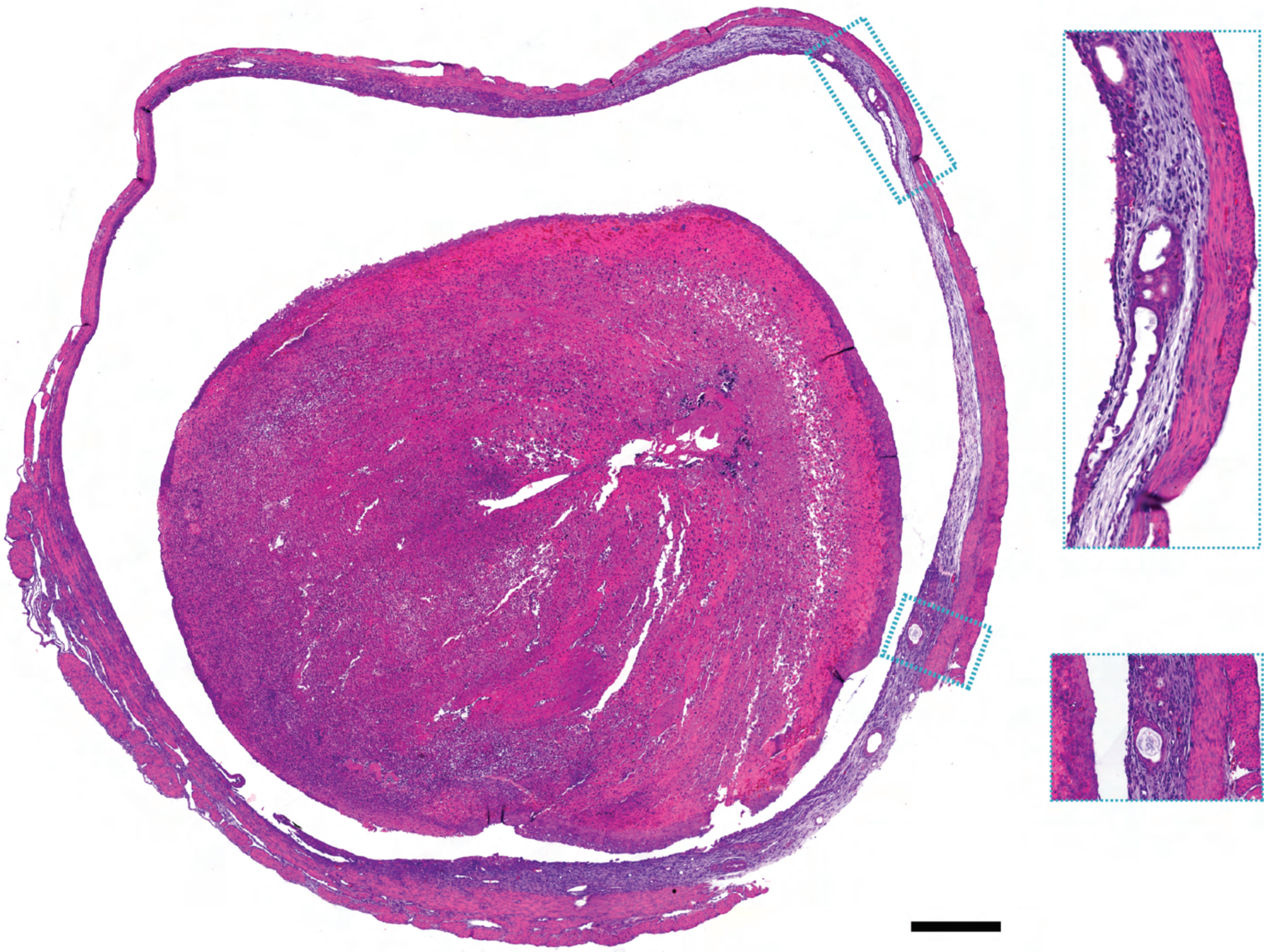

Figure S5. A basalis-like layer in X-Mens mice contains non-decidual endometrial tissue and glands.
H&E staining of an X-Mens uterus cross-section, harvested during menstruation. The inner functionalis-like layer is entirely detached from the outer basalis. Two inset images show glands and undifferentiated endometrial tissue residing in the basalis. Scale bar: 500μm.

A

timepoint	section thickness	#10X lanes	#cells	% spatially mapped
uninduced	2x30μm	2	16,841	%41.8
1 dpi early	20μm	1	3,058	%55.3
1 dpi late	20μm	2	13,000	%53.2
2 dpi	20μm	1	4,243	%60.2
3 dpi	20μm	2	12,418	%62.7
4 dpi (mens)	20μm	1	7,682	%73.6
secretory	20μm	3	33,179	%73.4
menstrual	20μm	3	36,346	%60.4

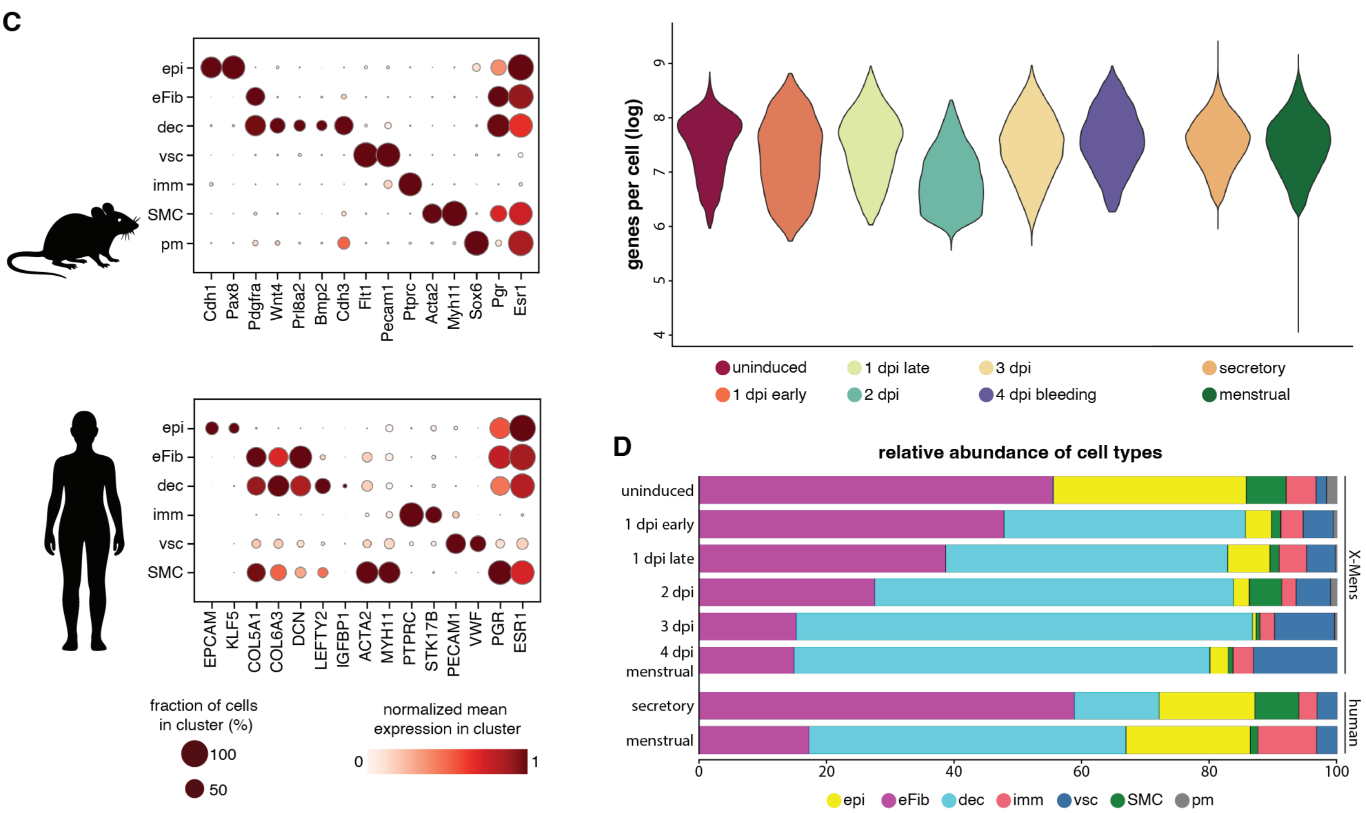

Figure S6. Quality control and cell type annotation for Slide-tags datasets from GsD X-Mens and human samples.
A. Table summarizing the number of 10X lanes, section thickness, number of cells, and percentage of cells spatially mapped for each Slide-tags sample. The first six samples are from X-Mens mice and the final 2 samples are human. **B.** Violin plots showing the distribution of log-transformed counts per cell and genes per cell for each sample in GsD X-Mens and human datasets. **C.** Dot plots depicting fraction and normalized mean expression of canonical marker genes for cell type in GsD X-Mens (upper) and human (lower) datasets. **D.** Bar plots showing the relative abundance of annotated cell types across all samples.

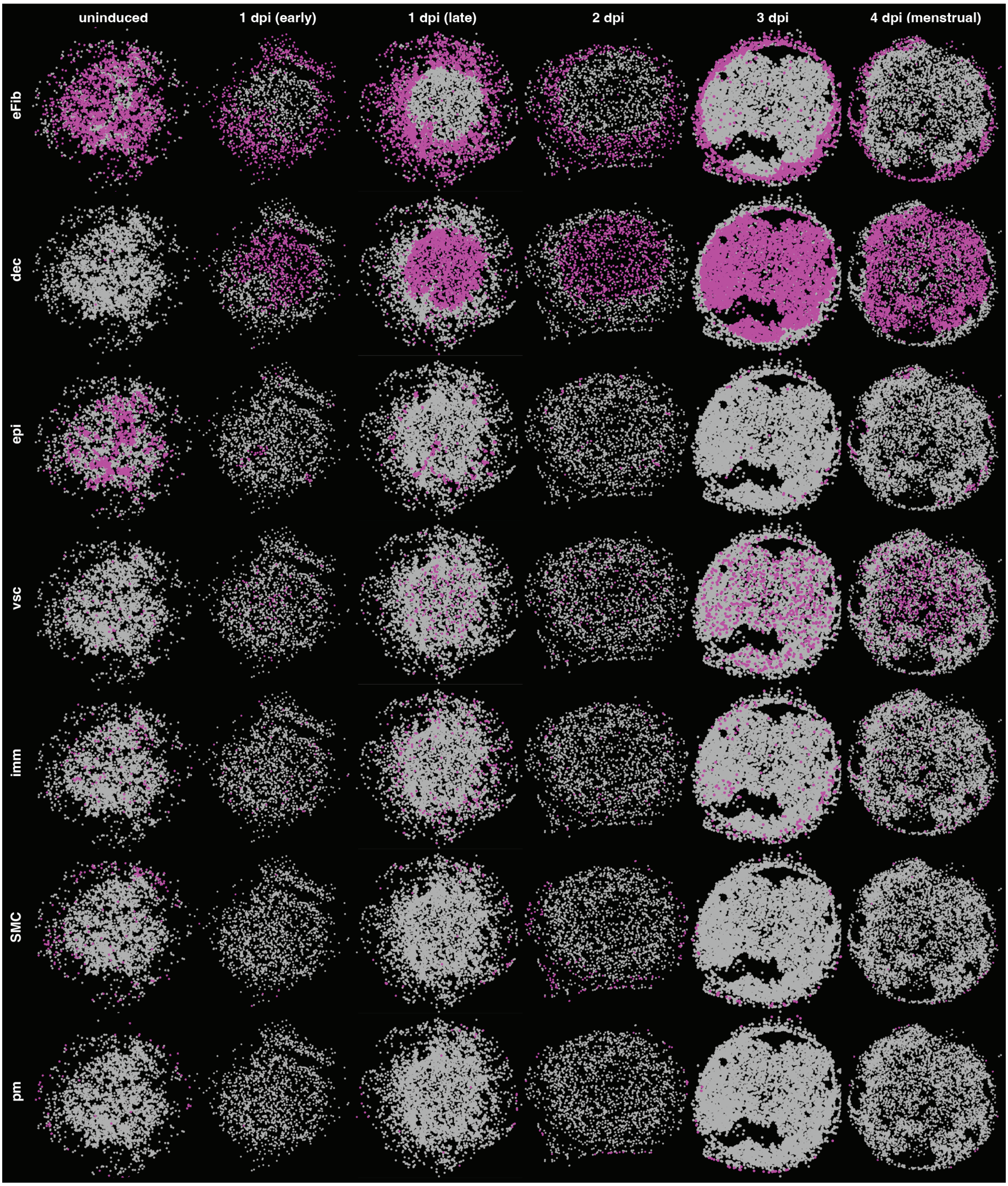

Figure S7. Spatial mapping of the cell types in GsD X-Mens Slide-tags samples.
Spatial distribution of major cell types (rows) in uterine sections from GsD X-Mens mice sampled at six stages (columns). Each panel highlights one cell type (magenta) overlaid on all other cells (gray). eFib: endometrial fibroblasts; dec: decidual cells; epi: epithelial cells; vsc: vasculature cells; imm: immune cells; SMC: smooth muscle cells: pm: perimetrium cells.

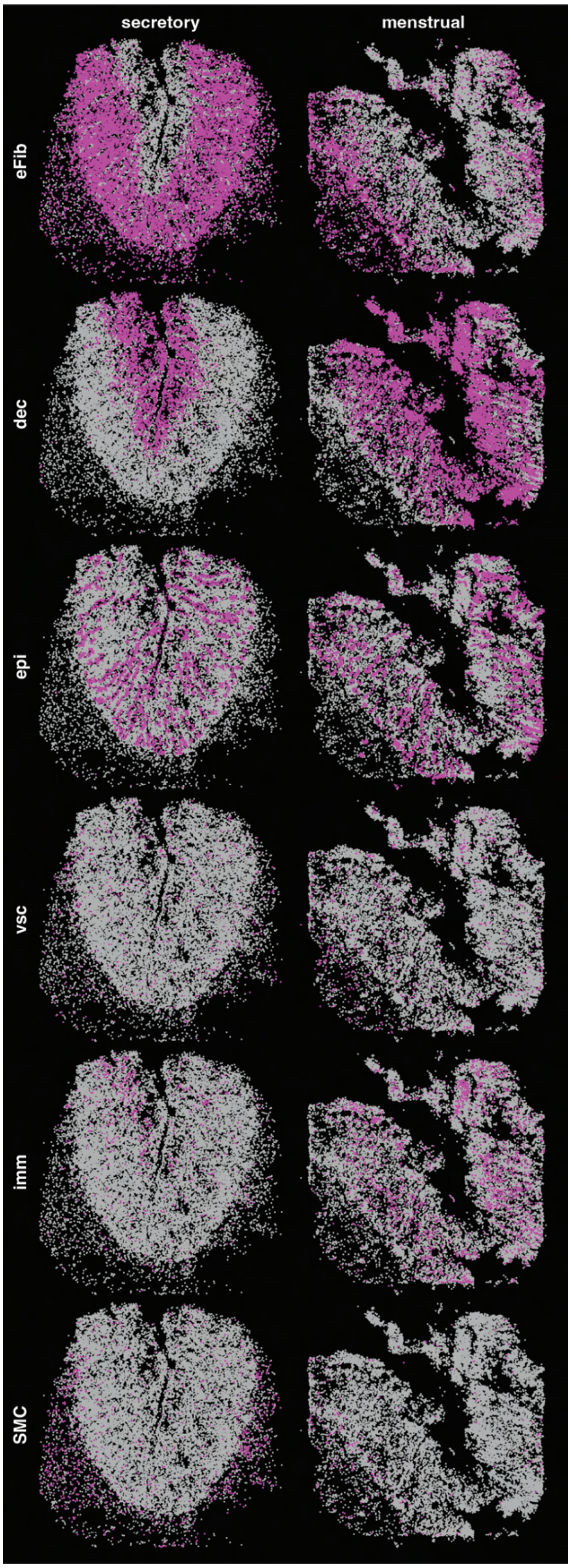

Figure S8. Spatial mapping of the cell types in human Slide-tags samples.
Spatial distribution of major cell types (rows) in uterine sections from human sampled at secretory and menstrual stages (columns). Each panel highlights one cell type (magenta) overlaid on all other cells (gray). eFib: endometrial fibroblasts; dec: decidual cells; epi: epithelial cells; vsc: vasculature cells; imm: immune cells; SMC: smooth muscle cells.

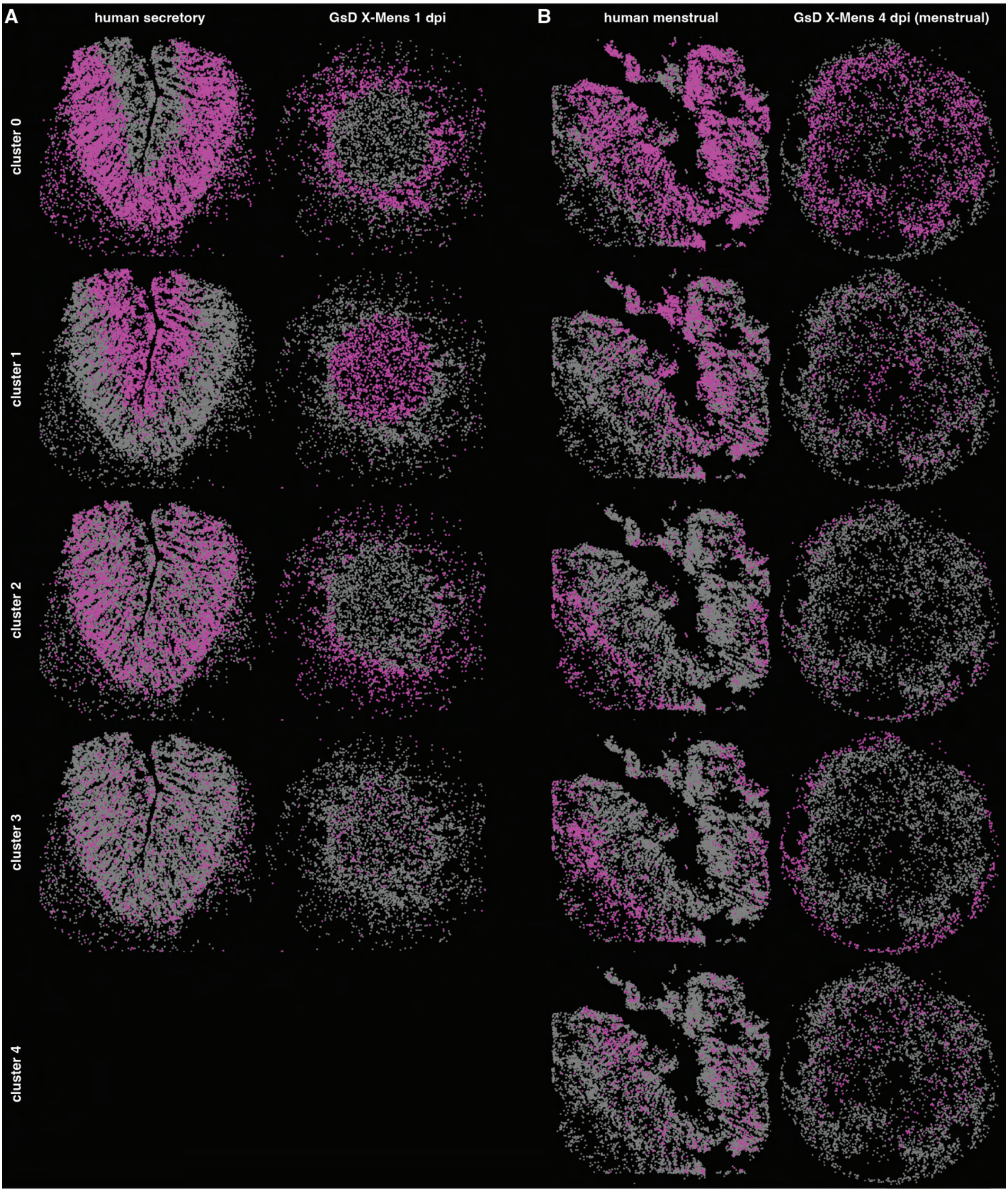

Figure S9. Spatial distribution of SAMap co-embedded cell clusters in human and GsD X-Mens endometrium.
A. Spatial mapping of clusters in the secretory (human) and 1 dpi (mouse) co-embedding object. **B.** Spatial mapping of clusters in the menstrual stage co-embedding object for both species. In each panel, the cluster of interest is shown in magenta overlaid on all other cells (gray).

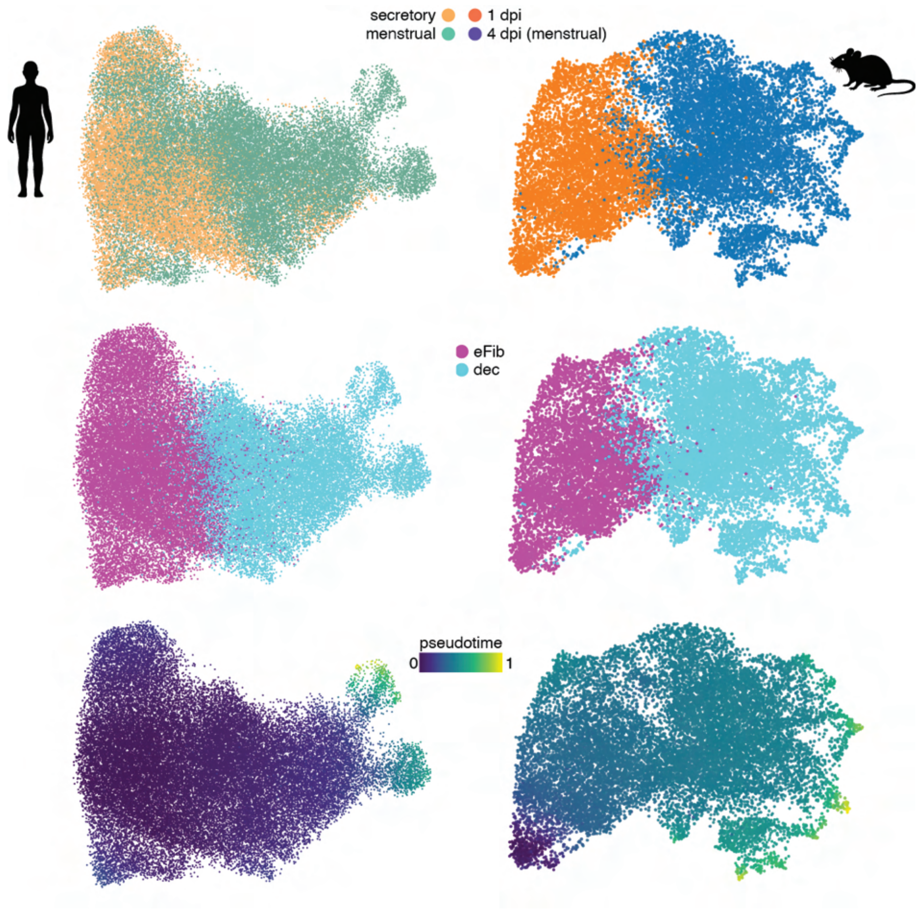

Figure S10. Pseudotime analysis of endometrial fibroblasts and decidual cells in human and GsD X-Mens decidualized and menstruating samples.
UMAP visualizations of single-nucleus transcriptomes from human (left) and GsD X-Mens mouse (right) endometrial fibroblasts (eFib) and decidual cells (dec). Rows display the same cells colored by: sample origin (top), cell type (middle), and pseudotime value (bottom).

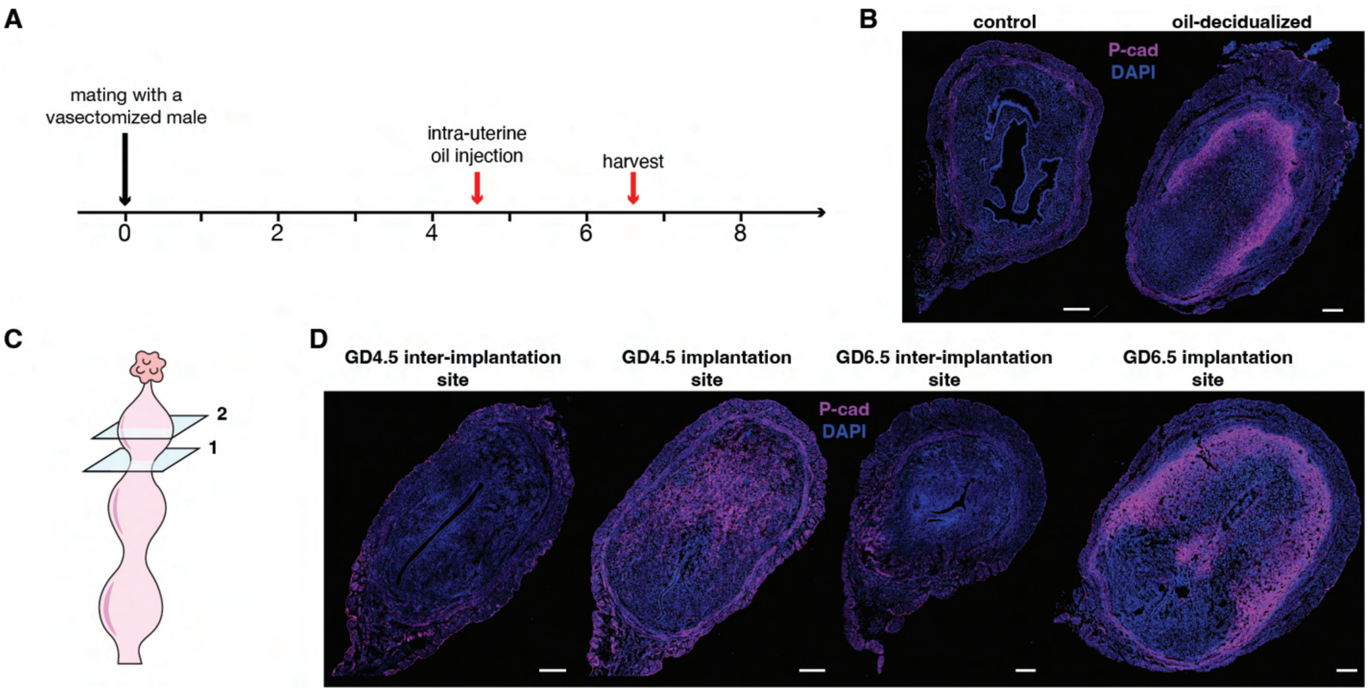

Figure S11. Cdh3 is a marker of mouse decidual cells.
A. Schematic timeline of the oil-mediated artificial decidualization protocol. Mice were mated with vasectomized males (arrow, day 0), received intrauterine oil injection (red arrow, day 4.5), and uteri were harvested at the indicated timepoint for analysis. **B.** Representative immunofluorescence images comparing Cdh3 expression (magenta) in control (non-decidualized) versus oil-decidualized uterine sections; nuclei are counterstained with DAPI (blue). **C.** Diagram illustrating the sectioning planes used for histological analysis of pregnant uterine horns. 1: inter-implantation site; 2: implantation site. **D.** Immunofluorescence images of pregnant uterine sections stained for Cdh3 (magenta), with corresponding stages indicated: inter-implantation and implantation sites at gestational day 4.5 (GD4.5) and 6.5. Scale bars: 200μm.

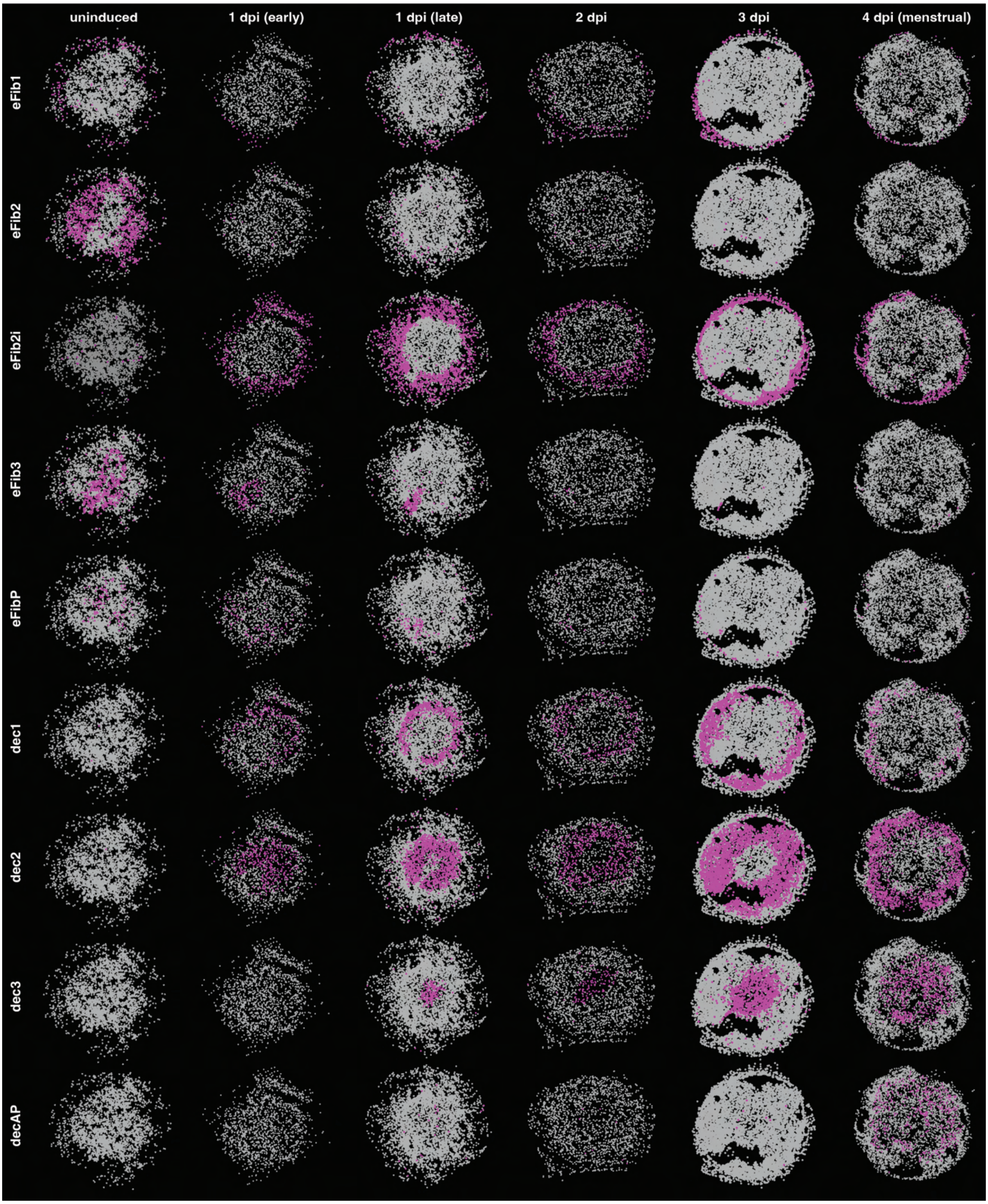

Figure S12. Spatial mapping of the endometrial fibroblast and decidual cell sub-types in GsD X-Mens Slide-tags samples.
Spatial distribution of endometrial fibroblast and decidual cell subtypes (rows) in uterine sections from GsD X-Mens mice sampled at six stages (columns). Each panel highlights one cell subtype (magenta) overlaid on all other cells (gray). eFib: endometrial fibroblasts; eFib2i: endometrial fibroblasts 2 induced; eFibP: proliferating endometrial fibroblasts; dec: decidual cells; decAP: apoptotic decidual cells.

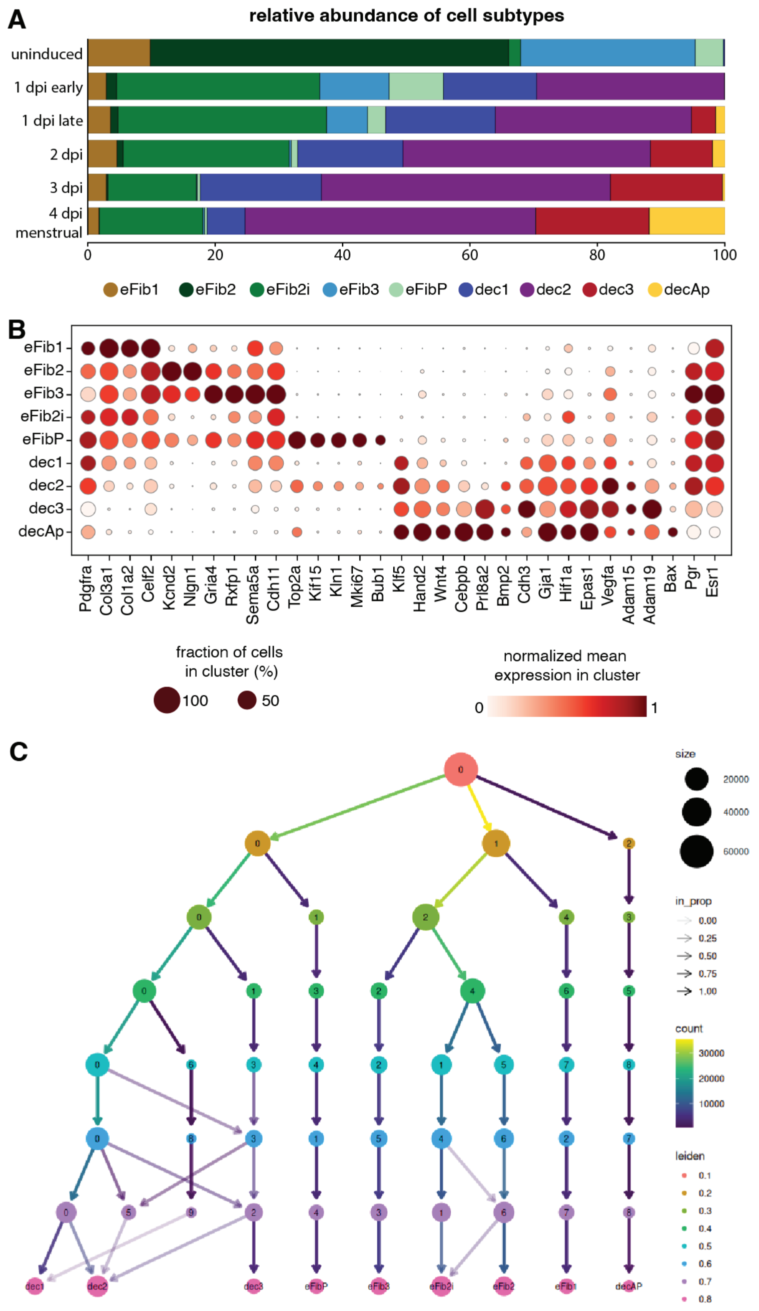

Figure S13. Characterization of fibroblast and decidual subtypes in GsD X-Mens endometrium.
A. Bar plots showing the relative abundance of endometrial fibroblast (eFib) and decidual (dec) subtypes at each sampled stage across the time course. **B.** Dot plot illustrating fraction and normalized mean expression of key marker genes, along with some cell cycle and hormone receptor genes, for each subtype. **C.** Clustree plot displaying clustering relationships and transitions between subtypes at varying Leiden resolutions; node size and color reflect cell count and clustering resolution, respectively. eFib: endometrial fibroblasts; eFib2i: endometrial fibroblasts 2 induced; eFibP: proliferating endometrial fibroblasts; dec: decidual cells; decAP: apoptotic decidual cells.

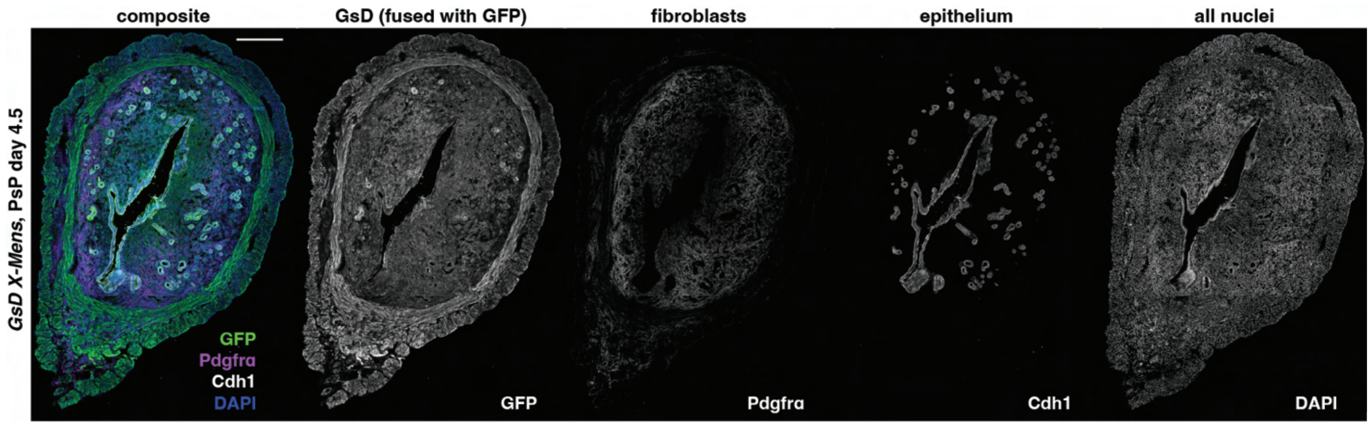

Figure S14. Expression pattern of GsD in the uterus at pseudopregnancy (PsP) day 4.5.
Representative cross-section of a GsD X-Mens mouse uterus stained for GFP (green; GsD-GFP fused protein), fibroblasts (Pdgfra, magenta), epithelium (Cdh1, white). Nuclei are counterstained with DAPI (blue). Scale bar: 400 μm.

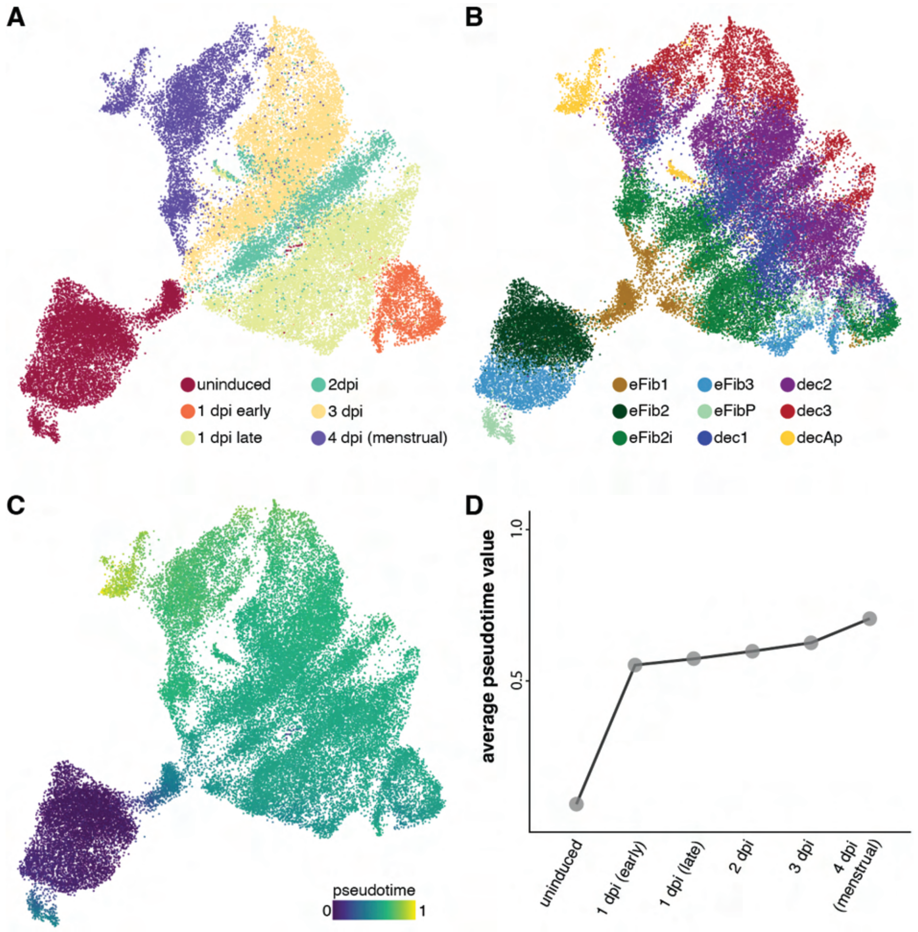

Figure S15. Pseudotime analysis of GsD X-Mens fibroblast and decidual cells.
UMAP visualization of fibroblast and decidual cells from all GsD X-Mens samples, shown: **A.** colored by sample, **B.** colored by cell subtype, **C.** colored by pseudotime value. **D.** Average pseudotime value for all cells in each sample. Dpi: days post-induction; eFib: endometrial fibroblasts; eFib2i: endometrial fibroblasts 2 induced; eFibP: proliferating endometrial fibroblasts; dec: decidual cells; decAP: apoptotic decidual cells.

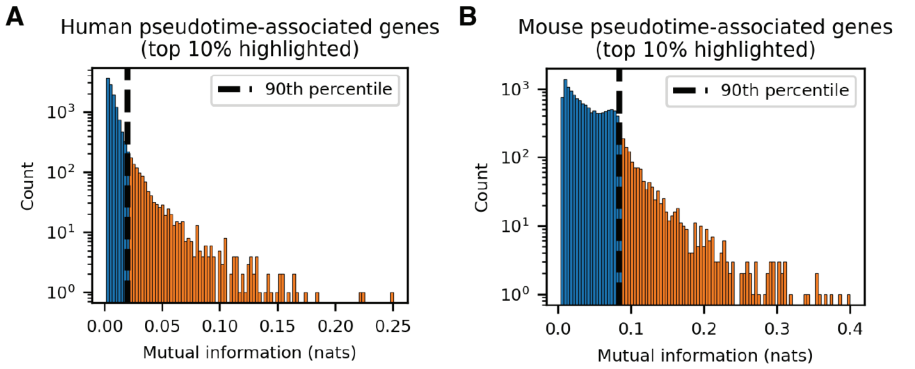

Figure S16. Selection of pseudotime-associated genes based on mutual information. Distribution of estimated mutual information values for **A.** human and **B.** mouse genes; the top 10% (highlighted in orange) are above the 90th percentile (dashed line).
