# Supplementary material for: Induction of menstruation in mice reveals the regulation of menstrual shedding
Source: bioRxiv. 2025 Oct 9:2025.10.08.681007. Preprint. [Version 1] doi: 10.1101/2025.10.08.681007 (PMC12668411; doi:10.1101/2025.10.08.681007)
Supplement: Supplement 3 [file NIHPP2025.10.08.681007v1-supplement-3.pdf]

bioRxiv preprint doi: https://doi.org/10.1101/2025.10.08.681007; this version posted October 9, 2025. The copyright holder for this preprint (which was not certified by peer review) is the author/funder, who has granted bioRxiv a license to display the preprint in perpetuity. It is made available under aCC-BY 4.0 International license.

Materials and methods

All material used in this study listed in Table S1.

Table S1: Materials used in this study.

Name	Vendor	catalog no
3-(trimethoxysilyl)propyl methacrylate	Sigma-Aldrich	440159
A83-01	Peprotech	9094360
acetic acid	Sigma-Aldrich	45754
Acrylamide/Bis-acrylamide	Biorad	1610144
Advanced DMEM	Thermo Fisher	12634010
ammonium persulfate	VWR	486
B27 supplement minus vitamin A	Thermo Fisher	12587010
bluing reagent	StatLab	SL102
bovine serum albumin (BSA)	Sigma-Aldrich	A3294
C-Chip Fuchs-Rosenthal disposable hemocytometer	INCYTO	DHC-F01-5
cell recovery medium	Corning	354253
clozapine-N-oxide dihydrochloride	Hello Bio	HB6149
clozapine-N-oxide dihydrochloride	MCE	HY-17366A
compound 21 dihydrochloride	Hello Bio	HB6124
compound 21 dihydrochloride	MCE	HY-100234A
spatially barcoded bead arrays	Curio	SK017
DAPI	Thermo Fisher	62248
deschloroclozapine dihydrochloride	Hello Bio	HB9126
deschloroclozapine dihydrochloride	MCE	HY-42110A
eosin	StatLab	SL101
ethanol 100%	StatLab	9500-1
ethanol 50%	StatLab	7050-1
ethanol 70%	StatLab	7070-1
ethanol 95%	StatLab	6900-1
ezDNase	Thermo Fisher	11766051
fetal bovine serum	VWR	76324-890
formaldehyde	EMS	15714-S
gentleMACS M tubes	Miltenyi Biotec	130-093-236
glucose	Sigma-Aldrich	49139
glutamine	Gibco	25030081
hematoxylin	StatLab	SL100
Hemoccult	Beckman Coulter	60151
HEPES (for organoid culture)	Cytiva	SH30237.01
HEPES (for Slide-tags)	VWR	511

Table S1 (continues): Materials used in this study.

Name	Vendor	catalog no
High-Def solution	StatLab	SL103
Histoclear II	EMS	64111-04
human EGF	Sigma-Aldrich	E9644-.5MG
human FGF-10	Peprotech	100-26-100UG
human HGF	Peprotech	100-39-50UG
human Noggin	Peprotech	120-10C
hydrophobic barrier pen	Vector Laboratories	H-4000
Kollidon VA64	BASF	50347977
Magnesium chloride	Invitrogen	AM9530G
Matrigel	Corning	356231
Matriplate	Fisher Scientific	NC9662693
Mifepristone	MCE	HY-13683
N-acetylcysteine	Sigma-Aldrich	A7250-5G
N,N,N′,N′-Tetramethylethylenediamine	Thermo Fisher	T9281
N2	Thermo Fisher	17502048
nicotinamide	Sigma-Aldrich	N3376-100G
normal donkey serum	Neuromics	SER004
nuclease-free water	Thermo Fisher	AM9937
OCT compound	Sakura Finetek	4583
paraffin	EMS	19280-01
PBS with azide	Santa Cruz	sc-296028
peanut oil	Sigma-Aldrich	P2144
PerfeCTa SYBR Green FastMix	Quanta Bio	95056
Permount mounting medium	EMS	17986-05
physiological saline	Hospira	NDC 0409-4888-10
positively charged glass slides	VWR	48311-703
potassium sulfate	VWR	BDH4618
primocin	InvivoGen	Ant-pm
ProLong Gold Antifade	Thermo Fisher	P36930
Qiashredder	Qiagen	79656
recombinant human R-spondin	R&D Systems	4645-RS-025/CF
recombinant mouse R-spondin	R&D Systems	7150-RS-050/CF
RNase inhibitor	Biosearch technologies	30281-1
RNeasy Mini Kit	Qiagen	74104
sodium deoxycholate	Sigma-Aldrich	30970
sodium sulfate	VWR	BDH9302
sucrose	Sigma-Aldrich	S0389
SuperScript IV First-Strand Synthesis	Thermo Fisher	18091200
Triton X-100	Sigma-Aldrich	X100-100ML
TrypLE Express	Gibco	12604039
xylenes	Sigma-Aldrich	534056
Y-27632 2HCl	Selleck Chemicals	S1049

bioRxiv preprint doi: https://doi.org/10.1101/2025.10.08.681007; this version posted October 9, 2025. The copyright holder for this preprint (which was not certified by peer review) is the author/funder, who has granted bioRxiv a license to display the preprint in perpetuity. It is made available under aCC-BY 4.0 International license.

Table S2: Mouse strains used in this study.

strain	full name	MGI ID	reference	source
B6	C57BL/6J	Jax: 000664	-	Jax
CD-1	CD-1 IGS	Charles River: 022	-	Charles River
Amhr2-Cre	B6.129(Cg)-Gt(ROSA)26Sortm4(ACTB-tdTomato,-EGFP)Luo/J	-	Jamin et al, 2002	Ron Chandler
Ltf-iCre	Ltftm1(icre)Tdku/J	Jax: 026030	Daikoku et al, 2014	Ron Chandler
GsD	Gt(ROSA)26Sortm1(CAG-Chrm3*/GFP,cAMPRE-luc)Berd	GsD	Akhmedov et al, 2017	Bruce Morgan
GqD	B6N;129-Tg(CAG-CHRM3*,-mCitrine)1Ute/J	GqD	Zhu et al, 2016	Jax
mTmG	B6.129(Cg)-Gt(ROSA)26Sortm4(ACTB-tdTomato,-EGFP)Luo/J	mTmG	Muzumdar et al, 2007	Jax

bioRxiv preprint doi: https://doi.org/10.1101/2025.10.08.681007; this version posted October 9, 2025. The copyright holder for this preprint (which was not certified by peer review) is the author/funder, who has granted bioRxiv a license to display the preprint in perpetuity. It is made available under aCC-BY 4.0 International license.

bioRxiv preprint doi: https://doi.org/10.1101/2025.10.08.681007; this version posted October 9, 2025. The copyright holder for this preprint (which was not certified by peer review) is the author/funder, who has granted bioRxiv a license to display the preprint in perpetuity. It is made available under aCC-BY 4.0 International license.

Table S3: Primers used in this study.

name	sequence
Mm_Actb-F	GGCTGTATTCCCCTCCATCG
Mm_Actb-R	CCAGTTGGTAACAATGCCATGT
Mm_Gapdh_F	CGTCCCGTAGACAAAATGGT
Mm_Gapdh-R	TCAATGAAGGGGTCGTTGAT
Mm_Prl8a2-F	GAGTCAACCTCACTTCTGGGC
Mm_Prl8a2-R	CTGAGCAGCCATTCTCTCCT

bioRxiv preprint doi: https://doi.org/10.1101/2025.10.08.681007; this version posted October 9, 2025. The copyright holder for this preprint (which was not certified by peer review) is the author/funder, who has granted bioRxiv a license to display the preprint in perpetuity. It is made available under aCC-BY 4.0 International license.

Table S4: Antibodies used in this study.

antibody	host organims	vendor	catalog #	RRID	dilution
Cdh1	rat	Invitrogen	13-1900	AB_2533005	1:1000
FoxA2	rabbit	Cell Signaling	8186	AB_10891055	1:200
GFP	chicken	Abcam	ab13970	AB_300798	1:1000
Cdh3	goat	R&D Systems	AF761	AB_355581	1:200
Pdgfrα	rabbit	Abcam	ab203491	AB_2892065	1:200
tdTomato	goat	Origene	AB8181-200	AB_3206272	1:1000
SMA	mouse	Sigma-Aldrich	C6198	AB_476856	1:1000
